# Label-Free Differentiation of Antimicrobial Resistance Groups Using Raman Spectroscopy

**DOI:** 10.1021/acs.analchem.5c03370

**Published:** 2026-02-23

**Authors:** Aikaterini Pistiki, Oleg Ryabchykov, Annette Wagenhaus, Thomas W. Bocklitz, Stefanie Deinhardt-Emmer, Bettina Löffler, Petra Rösch, Jürgen Popp

**Affiliations:** † Institute of Physical Chemistry and Abbe Center of Photonics, 9378 Friedrich Schiller University, Helmholtzweg 4, 07743 Jena, Germany; ‡ Leibniz-Institute of Photonic Technology Jena, Albert-Einstein-Str. 9, 07745 Jena, Germany; § InfectoGnostics Research Campus Jena, Center of Applied Research, 07743 Jena, Germany; ∥ Institute of Medical Microbiology, 39065 Jena University Hospital, 07747 Jena, Germany; ⊥ Cluster of Excellence Balance of the Microverse, Friedrich Schiller University Jena, 07743 Jena, Germany

## Abstract

Increasing antimicrobial resistance (AMR) has developed into an enormous health burden. Here, a systematic investigation was conducted to evaluate the discriminative performance of Raman spectroscopy between different resistance classes (Susceptible, ESBL, CRE, VRE, VSE) in common clinical isolates (*Escherichia coli*, *Klebsiella pneumoniae*, *Klebsiella oxytoca*, *Citrobacter freundii*, *Acinetobacter baumanii*, *Enterococcus faecium*). Two different Raman spectroscopic methods (UVRR in bulk and 785 nm excitation directly on the Petri dish) and four different machine learning algorithms (PCA-LDA, PLS-DA, PCA-SVM, PCA-RF) were tested aiming the application of a decision-tree using a 3-step approach composing of species classification, differentiation of susceptible from resistant strains within the species and differentiation of ESBL and CRE as AMR subclasses within the class of antibiotic-resistant strains. In species classification, the two Raman methods yield similar results in all applied models. When attempting the differentiation of susceptible vs resistant strains in the intraspecies level, 785 nm overall outperformed UVRR and PCA-SVM and PLS-DA provided higher discriminative power compared to PCA-LDA and PCA-RF. For the discrimination of ESBL vs CRE isolates UVRR was not suitable as a method and 785 nm excitation provided correct identification of all 9 strains when using PCA-SVM and PLS-DA, confirming stability over replicate-to-replicate variations. Raman spectra from 785 nm excitation directly on the Petri dish combined with PCA-SVM and PLS-DA are suitable for diagnostic application of Raman spectroscopy in hospital settings. These results are the first step of a long journey in the development of Raman spectroscopy for microbiological documentation and extraction of AMR-related information in infectious diseases.

## Graphical abstract

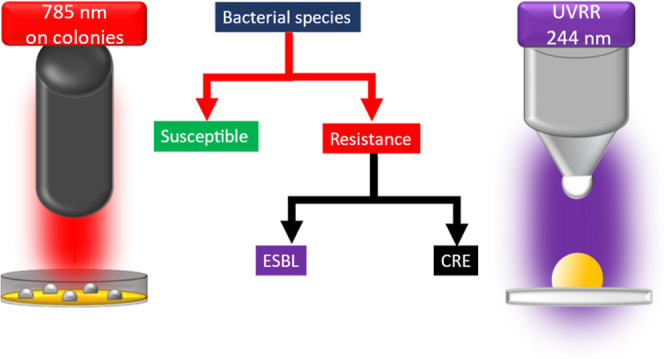

## Introduction

Antimicrobial resistance (AMR) derives as an evolutionary adjustment where microorganisms develop mechanisms to overcome the toxic effect of antibiotics on their survival and growth. The development of AMR-mechanisms followed the technological and industrial development of antimicrobials overtime and is amplified in environments with high antibiotic use as are health care units.^1^ One of the leading factors of AMR spread is the time-gap of several days between patient sampling and strain characterization that is caused by culture-based laboratory methods. These delays force physicians to administer empirical treatment, that is not always therapeutically appropriate and is often marked by the tendency of using last line antibiotics to ensure antimicrobial coverage and limit patient risk.^3,4^ This practice, however, contributes significantly to AMR increase^5^ and could not been overcome so far, mostly due to the high cost accompanying the fast and automated molecular techniques recently developed for clinical application.^6^ Thus, from a practical perspective and under current settings, any information on the strain’s AMR that physicians can get as early as possible, has a massive impact on patient outcomes and further on AMR spread.

The AMR of microorganisms is categorized based on their sensitivity to antibiotics in vitro and is classified into three categories namely susceptible, intermediate and resistant to specific antimicrobial agents.^7^ While several antibiotic agents can be effective against susceptible strains, the presence of resistance mechanisms, however, can make the strains untreatable with entire antibiotic groups, as are beta-lactams in extended spectrum beta-lactamase (ESBL) producing strains, or carbapenems in carbapenemase (CRE) producing strains that did develop after carbapenems were introduced to treat ESBLs. Information on pathogen’s classification into these AMR-categories at an early time-point, would serve as valuable guidance for physicians to select a more suitable treatment to counter the patient’s infection.

Metabolomic studies using spectroscopic techniques as Mass Spectrometry (MS) and Nuclear Magnetic Resonance (NMR) spectroscopy, have revealed that resistant strains have a different cell biochemistry than susceptible ones due to metabolic modification caused by the acquisition of resistant genes.^8,9^ Raman spectroscopy is a vibrational spectroscopic technique that captures the entire cell’s biochemical profile in form of a spectral fingerprint in a label-free way and it has been evidenced, that the metabolic modifications caused by resistant gene acquisition are reflected in the Raman spectra.^10^ This is displayed as differences in the intensities and shapes of the Raman bands providing a sum-spectrum that includes information on the overall biochemistry of the sample. Biomolecules carrying aromatic compounds show high absorption in the UV-range, thus in UV-resonance Raman spectroscopy (UVRR) signals from DNA and RNA bases and aromatic amino acids are intensively enhanced, dominating the spectrum and so by concretizing the spectral information in the sum-spectrum to these two specific sources. This allows the extraction of spectral information from the genotypic level of the strains, which is the biological source of species differences.

This technique is not capable to provide information on resistances to individual antibiotics, at least not without previous exposure to them^11^ due to superimposed signals of similar macromolecules in the same position of the spectrum. However, the obtained information can be used for discrimination of groups with similar biochemical characteristics, as are AMR-classes, allowing the fast and cost-effective nature of this technique to reveal clinically relevant information.

In previous, prove-of-concept studies, it was shown that application of different Raman spectroscopic techniques enabled the discrimination of nine laboratory transformed resistant *Escherichia coli* strains from their parental strain with an accuracy of 100%, linking the Raman signature to the genetic basis of the acquired resistance.^10^ In addition, multidrug resistant clinical *E. coli* strains^12^ as well as isogenic MSSA/MRSA strain pairs^13^ could be differentiated with high accuracy and within hours after strain isolation.

In the present study and for the first time, a systematic investigation, with six clinically important pathogens, namely *E. coli*, *Klebsiella pneumoniae*, *Klebsiella oxytoca*, *Acinetobacter baumanii*, *Citrobacter freundii*, and *Enterococcus faecium* belonging to five resistance classes, namely susceptible, ESBL and CRE as well as VRE and VSE was performed. The discrimination performance of two different Raman spectroscopic techniques (UV-resonance Raman spectroscopy on bulk bacterial sample and Raman fiber probe using 785 nm excitation directly on the Petri dish) and four different machine learning methods were evaluated, aiming to utilize a decision-tree for diagnostic application of Raman spectroscopy in hospital settings.

## Materials and Methods

### Bacterial Strains

Clinical isolates of six common pathogen species were selected. The strains derived from University of Thessaly hospital, Greece,^14,15^ a survey of CREs in a Pakistani Hospital^16^ and the Jena University Hospital. To reliably evaluate the classification models, the training data was cross-validated at the strain level, which is the highest available hierarchical level of the data set. Each strain was measured in three independent biological replicates (batches), to include any possible, sample-preparation-related biological variation into the data set. For the test data set a fourth independent batch was measured for one strain of each group. The species and number of strains belonging to each resistance-class for cross-validation (CV) and testing are shown in Table S1. Antimicrobial susceptibility testing was performed for all strains using the VITEK-2 system (Biomerieux, Marcy-l’Étoile, France). MIC results and resistance data based on EUCAST-breakpoints for all strains are shown in Table S2.

### Sample Preparation

The strains were stored at −80 °C and were transferred onto Tryptic Soy Broth (TSB) agar plates (Carl Roth, Karlsruhe, Germany). The agar plates were incubated overnight at 37 °C. A fresh preculture was prepared from the frozen stock for every measurement day. These precultivations were used for both Raman techniques. Cultivation and measurements of the batches were performed randomly to avoid the introduction of systematic bias into the data set.

For the Raman fiber probe, a loopful of biomass was transferred from the agar plate onto ø 75 mm Petri dishes made of stainless steel (Bochem, Weilburg, Germany), containing 8 mL TSB agar. Using stainless steel prevented the Raman signal from the Petri dish. The biomass was spread in straight lines onto the agar and incubated for 16 to 18 h at 37 °C.

For UVRR spectroscopy a few loopfuls of biomass were transferred from the agar plate into 20 mL TSB broth (Carl Roth) and were incubated for 1.5 h in a shaking incubator at 37 °C and 120 rpm to reach the exponential growth phase. Growth curves of all strains are shown in Figure S1. Due to the high biomass required for UVRR bulk measurements, the growth curves were performed using an OD_600_ of 0.5 as a starting point. This high concentration was used to better reflect the experimental conditions and ensure accurate indication of the time interval of the exponential growth phase, since dense inoculates were used in bacteria preparation for measurement. Since the device is located outside of a biosafety facility, the inoculum was then aliquoted into five separate 1.5 mL Eppendorf tubes followed by heat inactivation at 99 °C for 5 min for the Gram-negative species and for 10 min for *E. feacium*. Three consecutive washing steps with 1 mL deionized water (DI) were applied using centrifugation at 5,000*g* for 5 min (Minispin, Eppendorf, Hamburg, Germany).^12^ The bacterial pellet was then resuspended in 30 μL DI water, and all aliquots were placed onto separate fused-silica slides (B&M Optik GmbH, Germany) to air-dry at room temperature for ∼1 h. To verify the heat inactivation, 10 μL of each aliquot was plated onto TSB plates and incubation for 24 h at 37 °C. No growth could be detected for all tested strains.

### Raman Fiber Probe

Raman spectra were obtained from the bacterial colonies growing on the Petri dish using a Raman system (Kaiser Optical Systems, Ann Arbor, MI, USA) coupled with a 785 nm single-mode diode laser (Toptica, Gräfelfing, Germany) as previously described.^17^ To focus the laser onto the sample a Raman fiber probe (InPhotonics, Norwood, MA, USA) with a focal spot diameter of ∼50 μm and depth of field of ∼200 μm was used. The fiber delivered a laser power between 300 and 350 mW to the sample plane with a corresponding irradiance of 10^4^ W/cm.^2^ After passing a holographic transmissive grating, the scattered Raman signal was detected on a thermoelectrically cooled, back-illuminated, open-electrode charge coupled-device (CCD) chip (Andor, Northern Ireland), providing a spectral resolution of 4 cm^–1^. Each Raman spectrum was obtained from a single microbial colony with 10 s integration time and three accumulations. The minimal contribution of the TSB-agar in the bacterial spectra is shown in Figure S4. In the training data set, for each strain, three independent batches consisting of ∼20 spectra/batch and a total of 3512 spectra were collected. For testing, a fourth batch was measured for one strain per group and a total of 326 spectra were collected (Table S1).

### UV-Resonance Raman Spectroscopy (UVRR)

UVRR spectra were collected using a Raman microscope (HR800; Horiba Jobin-Yvon, Kyoto, Japan) with a focal length of 800 mm. The excitation wavelength of 244 nm was produced by doubling the frequency of the 488 nm line of an argon-ion laser (Coherent Innova 300; FReD) with a laser power of ∼20 mW, leading to ∼0.5 mW on the sample. The laser was directed and focused on the sample through a 40× antireflection-coated objective (LMU; numerical aperture, 0.5; UVB). Backscattered Raman light was collected through a 400 μm entrance slit into a 2.400-lines/mm grating and detected by a nitrogen-cooled CCD camera, leading to a spectral resolution of 2 cm^–1^. To avoid burning the sample, the sample stage was constantly rotated in a spiral manner during measurement using rotation speed of 30 rad/min. Each measurement consisted of ∼100 single spectra captured with 15 s integration. It has been previously shown that UV-irradiation of bacteria leads to protein damage after 5 min,^18^ however in our setup this phenomenon is avoided through the constant spiral rotation of the sample that results in the exposure of each sample spot to the laser for ms which is not enough to cause denaturation or burning of the sample. For the training data set three independent batches were measured for each strain and a total of 16.650 spectra were collected. For testing a fourth batch was obtained for one strain per group and a total of 1498 spectra were collected (Table S1).

### Data Analysis

Preprocessing and data analysis were performed using the RAMANMETRIX software, version 0.5.0 (https://ramanmetrix.eu)^19^ and included despiking based on one-dimensional Laplacian filter.^20^ The wavenumber calibration using reference spectra of 4-acetamidophenol (785 nm) or Teflon (UVRR) with a polynomial fit function with a degree of 3 or 2, respectively.^21^ These reference spectra were measured on each measurement day prior to the bacterial samples. Afterward, spectra were baseline corrected using a sensitive nonlinear iterative peak (SNIP)^22^ clipping algorithm with 40 iterations and a smoothing baseline of 10 followed by vector normalization. Spectra were then truncated to the relevant range of 400 to 1.800 cm^–1^.^23^ Pearson’s correlation with an average calibrated spectrum over the data set as a reference was applied, to filter out bad quality spectra and other outliers from the data set.^24^ For 785 nm excitation a correlation threshold of 0.725 after calibration and of 0.90 after preprocessing was set, with 89.66% of the spectra of the training data set and 93.86% of the test data set passing the filter (Table S1). For UVRR a correlation threshold of 0.97 after preprocessing was applied, with 92.88% of the spectra of the training data set and 88.45% of the test data set passing the filter. Prior to calculating the correlation after preprocessing, the reference spectrum was also subjected to the same preprocessing as the data set.

For each excitation wavelength, classification models were calculated to differentiate bacterial classes. The goal was to use a decision tree designed to be applicable in clinical settings. The models followed a 3-step approach aiming to gradually extract the biological information from each taxonomic level, while simultaneously keeping the comparisons as simple as possible to avoid unnecessary misclassifications due to complex statistics. At first, the bacterial species were differentiated at a species level in a 6-class model. Then for each species the resistant (ESBL and CRE) vs the susceptible strains followed by the subclasses ESBL vs CRE were classified by two 2-class models. For this, a set of supervised classifiers were used: (a) principal component analysis (PCA) combined with linear discriminant analysis (PCA-LDA), (b) PCA combined with support vector machine (PCA-SVM) using linear kernel, (c) PCA combined with random forest regression (PCA-RF) and (d) partial least-squares discriminant analysis (PLS-DA).^25^ These classification approaches were selected as they are among most commonly and successfully used in biomedical Raman spectral data analysis. These algorithms have implementations for different programming languages and make the analysis easily reproducible. All utilized classification methods, besides PLS-DA are combined with unsupervised dimension reduction by PCA to decrease influence of random noise onto classification models and avoid overfitting. The PLS-DA model is used without PCA as PLS is a dimension reduction technique itself. In each model the number of principal components (PCs) or latent variables (LVs) used was optimized based on the results of a leave-one-strain–out cross-validation (LOSOCV) as described by Guo et al.^26^ The PCA was calculated on the full training data set and used for the transformation of the test data. Since PCA is an unsupervised dimension reduction technique, model performance estimation should not have notable differences when performing PCA inside or outside the CV. However, PLS is a supervised approach, so it had to be recalculated for each cross-validation split to avoid data leakage.

A maximum number of 50 PCs (for PCA) or 15 LVs (for PLS) was set in all cases. After obtaining the classification prediction, a majority vote^12^ was taken to obtain strain-level prediction aiming to suppress in-sample heterogeneity and limit the effect of possible misclassifications on the final results. Balanced accuracy was calculated by averaging the sensitivity calculated for all classes. Finally, spectra were visualized using OriginPro, version 2018b (OriginLab Corporation, Northampton, USA).

## Results

### Species Classification

In this study two Raman spectroscopic techniques were applied in order to look at the cell’s biochemistry from two different perspectives. In the UVRR spectra, the signals from nucleic acid bases and aromatic amino acids are enhanced, capturing differences at the genetic/transcriptional level and partly on the protein level. When using the Raman fiber probe with 785 nm excitation, an overall profile of the cell’s biochemistry can be obtained, with signals deriving mainly from nucleic acids, proteins, lipids and carbohydrates, allowing good differentiation among bacterial species, including the highly related class of Enterobacteriaceae^27,28^ as well as epidemiological typing at the strain level.^29–32^ Also, limiting sample preparation via measurements directly on the Petri dish is of advantage. In addition, these two Raman methods use bulk analysis, meaning that Raman signals are collected from thousands of cells simultaneously. This provides averaging of Raman signals, reducing noise and minimization of intrasample heterogeneity, leading to more robust results.^33,34^

In Figure 1 mean Raman spectra of the bacterial species for both applied Raman methods and the positions of the significant Raman bands are shown. In the UVRR spectra (A), vibrational modes of DNA/RNA bases and aromatic amino acids can be detected, as are the ring vibrations of phenylalanine and tyrosine at 1610 cm^–1^,^35^ the stretching vibration along the axis of purines at 1481 cm^–1^ ^36^ and the ring breathing mode of phenylalanine at 1013 cm^–1^.^35,37^ In the 785 nm excitation (B) protein bands like amide I and amide III at 1658 and 1253 cm-1 ^35,38,39^ and CH_2_ deformation modes at 1421 cm^–1^ ^39^ as well as nucleic acid bands as the O--P--O stretching DNA backbone signal at 782 cm^–1^ ^35,40^ can be found. A detailed band assignment is shown in Tables S3 and S4.

**Figure 1:**
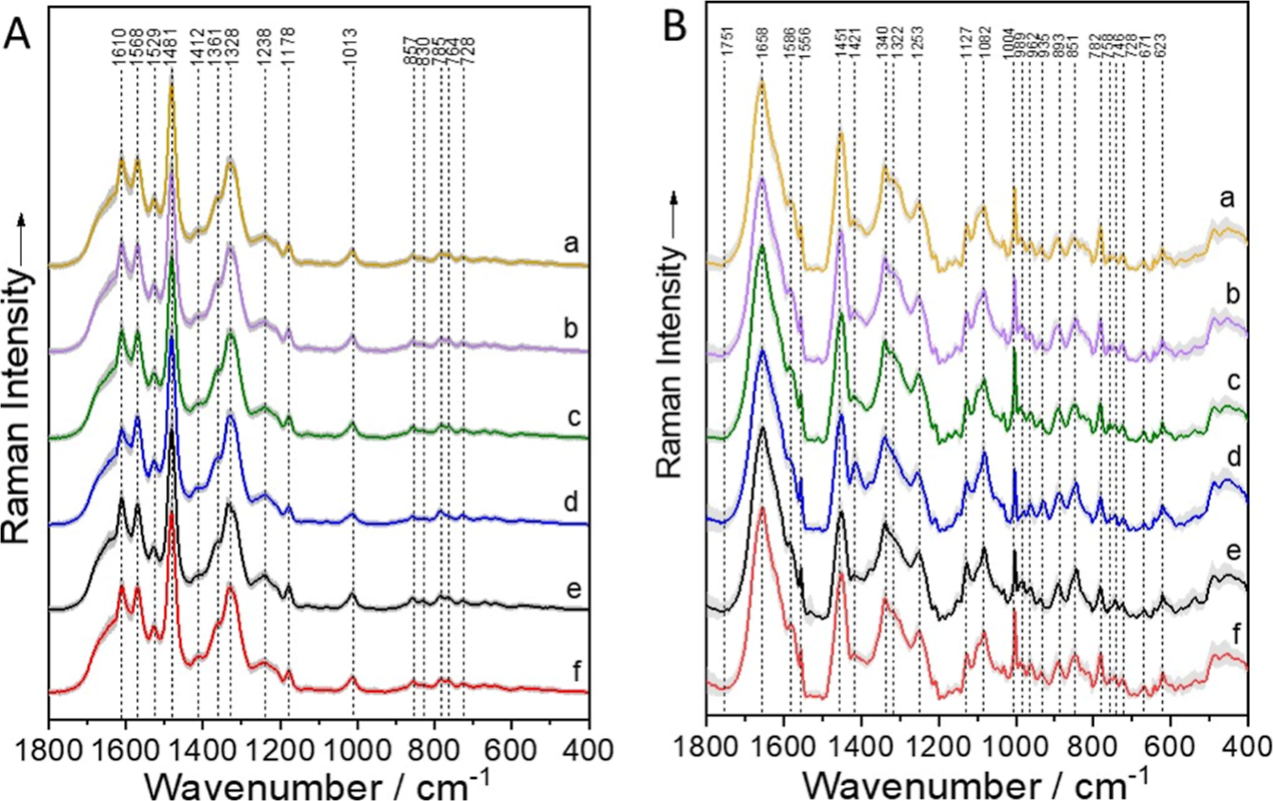
Mean Raman spectra ± SD of the investigated bacterial species measured with A. UVRR spectroscopy and B. Raman fiber probe. Analysis includes all strains of the species: a. *K. pneumoniae*, b. *K. oxytoca*, c. *E. coli*, d. *E. faecium*, e. *A. baumanii*, f. *C. freundii*.

The classification results of the calculated models at the taxonomic level of the species are shown in Table 1. The confusion matrices and 95% confidence intervals (CI) of sensitivity for all models are presented in Table S5 for the training models and CIs for test data are shown in Table S6, with the intervals estimated from the Beta posterior distribution. The confusion matrix of all models is shown in Table S5, the PCA-LDA coefficients for each bacterial species are shown in Figure S2.

**Table 1:** Cross-Validation and Test Results of Bacterial Species for UVRR Spectroscopy and Raman Fibre Probe

	PCA-LDA	PCA-SVM	PLS-DA	PCA-RF
Cross-Validation (Balanced Accuracy/%)				
UVRR	81.4	81.9	76.7	56.1
785 nm	81.8	88.3	88.3	72.5
Test (Balanced Accuracy/%)				
UVRR	66.7	72.2	58.3	58.3
785 nm	100	94.4	94.4	86.1

When comparing the two Raman methods classification accuracies of these 6-class models were higher for 785 nm excitation in both cross-validation and testing. Also, PCA-LDA, PCA-SVM and PLS-DA show similar discrimination power in cross-validation. In testing, lower accuracies were yielded in the UVRR, indicating large batch variations. The test performance for the 785 nm excitation was higher, as expected for lower batch variations when the same strains are included in training and test data sets.

For both, cross-validation and testing, PCA-RF displayed poor performance in UVRR and fair performance in 785 nm excitation, providing the lowest classification accuracies of all.

### Discrimination between Resistance Classes

Here, the first step was the differentiation of susceptible strains from the resistant ones. For this, the ESBL and CRE strains were grouped together into one class of antibiotic-resistant strains and compared to the susceptible strains. Results are summarized in Table 2. The confusion matrices and 95% confidence intervals (CI) of sensitivity for all models are presented in Table S5 for training and in Table S7 for test data. The mean, normalized spectra of all classes compared with the important Raman bands marked, are shown in Figure S3. The differences in the Raman spectra are difficult to see by the naked eye and require machine learning models for their detailed visualization, however, the major differences in the presence/absence of specific Raman bands and their intensity can be seen when observing the mean spectra of two or more groups in comparison. These differences reflect the biochemical differences present in the bacterial cells of the different strain classes. In most cases, the balanced accuracies obtained through cross-validation were similar for both Raman methods, with the 785 nm excitation method performing slightly better overall. Concerning the statistical methods, PCA-RF provided in general the worst discriminative ability of all in both cross-validation and testing. In the cross validation of all other methods, with some exceptions such as PLS-DA for *K. oxytoca* and *E. faecium* in UVRR and PCA-SVM in *C. freundii* in 785 nm excitation, the classification power followed similar trends in each species. Interestingly, the differentiation of the susceptible and resistant strains varied significantly among the different species, with balanced accuracies ranging from below 50% to 100%. In UVRR, sufficiently good results were obtained for *A. baumanii* and *E. coli*, fair results for *K. oxytoca*, while *E. faecium* and *K. pneumoniae* showed no discrimination potential in any of the models. In the 785 nm excitation, good cross validation results were obtained for *A. baumanii*, *E. coli* and *K. pneumoniae*, and a fair performance for *C. freundii* and *E. faecium*. These variances indicate that each species displays a different discriminative potential in differentiating susceptible and resistant strains.

**Table 2:** Cross-Validation Results of Susceptible vs Resistant Strains for Each Bacterial Species

		PCA-LDA	PCA-SVM	PLS-DA	PCA-RF
Cross-Validation (Balanced Accuracy/%)					
UVRR	*A. baumanii*	83.3	66.7	75	66.7
	*C. freundii*	66.7	50	50	0
	*E. faecium*	50	62.5	37.5	12.5
	*E. coli*	100	100	100	75
	*K. oxytoca*	66.7	66.7	16.7	0
	*K. pneumoniae*	55	25	40	50
785 nm	*A. baumanii*	75	66.7	58.3	66.7
	C. freundii	66.7	83.3	66.7	33.3
	*E. faecium*	75	62.5	62.5	50
	*E. coli*	93.8	75	87.5	100
	*K. oxytoca*	66.7	66.7	66.7	16.7
	*K. pneumoniae*	85	90	95	50

When considering cross-validation and testing the overall best performance of susceptibility detection was provided by PCA-SVM and PLS-DA. Lower performance of PCA-LDA analysis could be because the variance within the susceptible class is smaller than within the class of antibiotic resistant strains (combined ESBL and CRE), which contradicts assumptions of LDA algorithm. Other utilized supervised models, such as PLS, SVM, and RF do not assume that the classes have the same variance, so they should be more suitable in this case. Here, however, PCA-RF might underperform in cross-validation and testing due to overfitting. Although merging groups (ESBL, CRE) introduce class imbalance, investigation of its effect is a complex issue which is outside of the scope of this manuscript.

### Discrimination within the AMR Subclasses

Next, the differentiation of the ESBL and CRE strains composing the AMR subclass was attempted. Results are shown in Table 3. The confusion matrices and 95% confidence intervals (CI) of sensitivity for all models are presented in Table S5 for the training models and CIs for test data are shown in Table S8.

**Table 3:** Cross-Validation Results of ESBL vs CRE Strains for Each Bacterial Species

		PCA-LDA	PCA-SVM	PLS-DA	PCA-RF
Cross-Validation (Balanced Accuracy/%)					
UVRR	*A. baumanii*	33.3	33.3	50	16.7
	*E. coli*	37.5	50	25	0
	*K. pneumoniae*	30	30	10	50
785 nm	*A. baumanii*	33.3	50	33.3	50
	*E. coli*	62.5	50	62.5	62.5
	*K. pneumoniae*	80	70	70	80

Results of UVRR show that this method is unsuitable for the discrimination between ESBL and CRE strains, since the yielded cross-validation accuracy did not exceed 50%. With 785 nm excitation, accuracies were higher for cross validation, reaching up to 80% for *K. pneumoniae* and providing consistently good prediction in testing (Table S6) in almost all models, indicating consistent batch comparability. Model performance yielded similar balanced accuracies in all statistical methods thus, no definitive conclusion can be made on which performs best.

## Discussion

The present study demonstrates that by obtaining Raman spectra with the Raman fiber probe (785 nm), a clinically applicable decision-tree can be utilized. Using a 3-step approach, composing of species classification, followed by differentiation of susceptible from resistant strains within the species and finally differentiation of ESBL and CRE, clinically relevant information can be extracted from the Raman spectra in a fast and label-free way. For the Gram-negative isolates fair to good balanced accuracies were yielded, despite the high variations among the models. Adequate discriminative power for ESBL and CRE could only be achieved for *K. pneumoniae*, where similar balanced accuracies were yielded for all statistical methods, except PCA-RF that generally performed poor in all comparisons. In *E. faecium* discrimination of VSE and VRE could not be achieved with any of the applied Raman and statistical methods. UVRR performed poorly for the resistance classes but similar to 785 nm excitation for the species classification.

In this study, the variation between the bacterial strains is biological, deriving from interspecies biochemical differences as well as differences in the supragenome and the cell metabolism at the intraspecies level. When classifying bacteria species, the differences between the species are large enough to overcome the differences between their individual strains and therefore models perform well in cross-validation and testing.

When classifying the antimicrobial resistance groups at a strain level, the differences are small, and the available statistical methods display different advantages and disadvantages. Linear methods such as PCA-LDA and PLS-DA are more stable and are performing better in testing 2-class problems than more complex methods such PCA-SVM and PCA-RF. However, this outperformance is eliminated when the fine line between the training model performance and overfitting is crossed, that can be due to a high number of used PCA or PLS components. The utilized LOSOCV approach mitigates overestimation of cross-validation performance due to memorizing specific strains. This is reflected by low performance for PCA-RF due to random forest being a very strong classifier which is prone to memorizing instead of generalizing on smaller data sets, which is not the case for the other utilized models: PCA-LDA, PCA-SVM (linear kernel) and PLS-DA. Among the classification methods, PCA-SVM and PLS-DA performed similarly and best among the resistance classes. The slightly worse performance of PCA-LDA may be attributed to the assumptions of LDA regarding data distribution. LDA assumes that the data within each class is normally distributed and that these distributions are the same for all classes. In this analysis, ESBL and CRE strains were grouped together for the species *Acinetobacter baumannii*, *E. coli*, and *K. pneumoniae*, forming a single class of antibiotic-resistant strains. Having multiple subgroups in each class might not correspond to the normal distribution assumption. More importantly, it introduces higher variations in interclass resistance group variance compared to the interclass variance of susceptible strains. This difference in variances could not be compensated by the linear decomposition of PCA-LDA, which led to lower accuracy, especially in testing. These assumptions do not apply to SVM and PLS-DA, making them more suitable for cases with different interclass variances.

In the present data set, cross-validated and test accuracies may resemble differences in performance which we can attributed to the validation scheme necessitated by the limited amount of data. To mitigate the risk of overestimating model performance due to models memorizing specific strains, we employed LOSOCV. However, this cross-validation approach does not reliably reflect the models’ sensitivity to batch-to-batch variations. When the test batch is similar to the training batches, test performance is expected to increase, as the training data already includes the analyzed strains. In contrast, high batch-to-batch variation would lead to a decrease in test performance.

It has to be mentioned that only one strain per group was used for testing in ESBL vs CRE and some susceptible vs resistant cases, causing large confidence intervals in classification accuracies, making the comparison of the individual test accuracy values unreliable. However, an overall trend (Table S7) suggests consistently better test predictions for 785 nm excitation Raman data in comparison to UVRR.

Bacterial species classification has been successfully performed in the past using Raman spectroscopy^23,41–45^ with similar results as in the present study. In particular, when UVRR was applied onto 20 clinical isolates from urinary-tract infection (UTI), including the highly related group of Enterobacteriacae, a clear clustering of the species was achieved.^46^ UVRR could also successfully differentiate strains of the phylogenetic very closely related genera Bacillus and Brevibacillus, in a similar manner as 16S rDNA analysis.^47^ Here, however, species classification is the first step of a decision-tree and is prerequisite to exclude the data of the species that are not used in the next analysis step. Once the species is known, spectral information concerning the strains’ AMR can be extracted through comparisons with spectra from strains of the same species and with known AMR. From a technical perspective, this is done to simplify the process and ensure high accuracy since in data analysis only relevant comparisons are performed and the unnecessary data in the database are filtered out in a case-specific manner. This is also necessary because of the high similarities in the Raman spectra captured at an intraspecies level and the minor impact resistance genes have to the entire cell’s biochemistry as shown in Figures S2 and S3. From a clinical perspective, it provides all the information required by physicians for therapeutical decision making. In pathogenic strains AMR derives from two separate sources. One is the development or uptake of resistance genes, transforming a susceptible strain to a resistant one. The other source is the natural resistance present in specific species, deriving from their endogenous physiological characteristics that are not compatible with the mechanism of action of certain antimicrobial agents. Thus, the knowledge on the strains’ species and their AMR-classes are complementary, allowing treatment concretization and de-escalation at an early time point when using Raman spectroscopy. Molecular techniques as PCR-based methods, have similar advantages in speed, their high costs for devices and consumables, however, especially when using multiplex approaches, is often prohibitive for large scale use or for small hospital units with limited budged options. The Raman approach overcomes this issue since no further consumables are required for testing.

The current data show that the overall biochemical profile captured by 785 nm excitation using the Raman fiber probe, reflects better the minor biochemical differences between the AMR classes and its minimal requirements in sample preparation is more suitable for daily laboratory routine. UVRR on the other hand does not perform as expected. Even though the AMR arises through genetic modifications, it is shown here that their impact is too small to produce spectral differences that are large enough to provide high classification accuracies. The effect of AMR genes on overall cell biochemistry, however, results in larger alteration, allowing better classification performance using 785 nm excitation.

Metabolomics analysis has extensively defined the alterations generating the differences between the resistance classes captured in the Raman spectra. In *E. coli* it has been shown that chromosome mediated colistin resistance carried a fitness cost, in contrast to plasmid-mediated resistance that showed similar fitness as susceptible strains.^48^ Also, mcr-1 generated colistin resistance displayed decreased growth rates and cell viability, changes in membrane permeability that lead to decreasing resistance to hydrophobic antibiotics, and changes in cellular as well as colony morphology.^49^

When comparing a laboratory derived colistin resistant *A. baumanii* strain to its susceptible parental strain, significantly lower growth rates were detected, nearly 25% of metabolites were more abundant and growth medium-deriving peptides were highly enriched, indicating increased accumulation of medium components. Also, enrichment of short-chain fatty acids and short-chain lysophospholipids were found. These changes were not detected when using a clinical strain pair, isolated from a patient before and after colistin treatment, where generally fewer metabolite differences were detected. However, common features were captured in the laboratory and clinical strain pairs with tricarboxylic acid (TCA)-cycle related metabolic changes and peptidoglycan biosynthesis intermediates being of lower relative abundance in colistin-resistance.^50^ Aye et al., performed metabolomics on three clinical multidrug resistant (MDR) *K. pneumoniae* strain pairs, isolated from the gut of patients before and after colistin treatment. The resistant strains showed lower levels of several fatty acids, metabolites of the central carbon metabolism as well as several components of the pentose phosphate pathway (PPP) and purine and pyrimidine biosynthesis. In addition, lower carbon flow into the TCA-cycle was detected, influencing cell energy production similar as for *A. baumanii* mentioned above. Furthermore, they displayed lower nucleotide pools, a perturbed amino acid metabolism, especially for histidine. Also, depletion of amino-sugar metabolites related to cell wall biosynthesis, increased level of Kdo, increased intracellular levels of phosphatidylethanolamine (PE) and phospatidylglycerol (PG) phospholipids that when combined with the lipid A modification and changes in the PPP indicate that a cell envelop remodelling could be evidenced in some of the resistant strains.^51^

The above-mentioned studies compared strain pairs of similar genetical background and with one specific resistance. The present study uses MDR clinical isolates with the differences between the strains and the resistance groups surpassing these controlled settings and despite the high biological variance introduced into the data set, the high sensitivity of Raman spectroscopy displays immense discriminative power. It has to be mentioned that in this study, the very edge of Raman spectroscopy’s abilities is exploited, and this is reflected in the results where large differences in yielded accuracies were detected among the species when AMR profiling was attempted. The differentiation of VRE and VSE as well as ESBS from CRE in *A. baumanii* and *E. coli* did not provide high accuracies in cross-validation. For these species the analysis cannot be conducted beyond the species classification level and further resistance-related information cannot be extracted. In these cases, the information provided to physicians to use for their therapeutical decision making is more limited. This, however, is already more than currently available 24 h after sampling and is valuable information that, when combined with patients’ history, can guide therapeutical decision making and allow treatment adjustment and de-escalation at an early time point in some patient groups. It has to be considered that these issues could be overcome when enlarging the data sets by including higher number of supragenome variations into each class and limiting confidence intervals, especially in testing. This can be done by performing individual and combined studies on different species with large number of strains, considering that the differences in these strains are often as minimal as one SNP within the entire genome.

In addition to the pathogen species used in this study, *Pseudomonas aeruginosa* and *Staphylococcus aureus* are also common pathogens of high clinical importance. These two species could not be included into the data set due to features in their Raman spectrum that do not allow further analysis after species classification when measured with 785 nm excitation. In the Raman spectrum of *S. aureus*, the dominant bands of the carotenoid staphyloxanthin, suppress all other signals of the cell spectrum.^13^ The Raman spectrum of *P. aeruginosa* contains a lot of fluorescence due to the excretion of pyoverdine and pyocyanin into the culture medium that overpowers the Raman signals.^28^

## Conclusions

The present study is the first attempt to extract AMR-related information, from several bacteria species in a large data set, with a systematic approach that can be applicable in form of a clinically relevant decision tree. Conclusions could be drawn on the investigated parameters, namely Raman spectroscopic method and statistical analysis, however, it was also seen that the high variance among the strains of a species influences result accuracies, showing that further research is required, with larger data sets. Future investigations should focus on 785 nm excitation and use the advantages in terms of costs, speed and simplicity provided by the Raman fiber probe directly on the Petri dish. Also, for data analysis, PCA-SVM and PLS-DA should be preferred to better compensate the high interspecies variance when classifying strains with such small differences as are antimicrobial resistances. This approach could provide physicians with AMR related information within the first 24 h after sampling, allowing treatment concretization and de-escalation if required.

## Supplementary Material



## Notes

Number of used strains per class, testing results, PCA-LDA coefficients, confusion matrixes, tentative band assignments, growth curves, MIC results (PDF)

## Abbreviations

MS: mass spectrometry
NMR: nuclear magnetic resonance spectrometry
MSSA: methicillin-susceptible *Staphylococcus aureus*
MRSA: methicillin-resistant *Staphylococcus aureus*
ESBL: extended Spectrum Beta-lactamase
CRE: carbapenem-resistant *Enterobacteriaceae*
VRE: vancomycin resistant *Enterococcus*
VSE: vancomycin susceptible *Enterococcus*
CV: cross-validation
MIC: minimal inhibitory concentration
EUCAST: European Committee on Antimicrobial Susceptibility Testing
TSB: tryptic soy blood agar
DI: deionized water
UVRR: UV-resonance Raman Spectroscopy
CCD: charge coupled-device
SNIP: sensitive nonlinear iterative peak
PCA: principal-component analysis
LDA: linear discriminant analysis
SVM: support vector machine
RF: random forest regression
PLS-DA: partial least squares discriminant analysis
PC: principal components
LV: latent variables
LOSOCV: leave-one-strain–out cross-validation
TCA: tricarboxylic acid-cycle
MDR: multi-drug resistant
PPP: pentose phosphate pathway
PE: phosphatidylethanolamine
PG: phospatidylglycerol
