# Supplementary material for: Label-Free Differentiation of Antimicrobial Resistance Groups Using Raman Spectroscopy
Source: Anal Chem. 2026 Feb 23;98(9):6523–31. doi: 10.1021/acs.analchem.5c03370 (PMC12980480; doi:10.1021/acs.analchem.5c03370)
Supplement: Supplementary file 1 [file ac5c03370_si_001.pdf]

Supporting Information

Label-free differentiation of antimicrobial resistance groups using Raman spectroscopy

Aikaterini Pistiki[a,b,c], Oleg Ryabchykov[a,b], Annette Wagenhaus[a,b,c], Thomas W Bocklitz[a,b], Stefanie Deinhardt-Emmer[d], Bettina Löffler[c,d], Petra Rösch[a,c,*], Jürgen Popp[a,b,c,e]

[a] Institute of Physical Chemistry and Abbe Center of Photonics, Friedrich Schiller University, Helmholtzweg 4, 07743 Jena, Germany

[b] Leibniz-Institute of Photonic Technology Jena, Albert-Einstein-Str. 9, 07745 Jena, Germany

[c] InfectoGnostics Research Campus Jena, Center of Applied Research, 07743 Jena, Germany

[d] Institute of Medical Microbiology, Jena University Hospital, 07747 Jena, Germany

[e] Cluster of Excellence Balance of the Microverse, Friedrich Schiller University Jena, 07743 Jena, Germany

Corresponding Author:

*E-Mail: petra.roesch@uni-jena.de. Phone: +49-36419-48381. Fax: +49-36419-48302.

TABLE OF CONTENT:

Figure S1. Grow curves of all used strains. To obtain the grow curves, a bacteria inoculum was prepared with an OD 600 of 0.5. This was then incubated in a shaking incubator and bacteria growth was monitored by obtaining the cultures OD 600 every 30 minutes.

Figure S2. PCA-LDA coefficients for species classification based on Raman spectra using UVRR and 785 nm excitation. Each plot visualizes the coefficients used to identify a specific species among the others.

Figure S3A. Mean Raman spectra of 785 nm excitation directly on the Petri dish and band positions of all bacterial species. Average spectra of all measured strains in the susceptible and resistant groups are shown in comparison

Figure S3B. Mean Raman spectra of 785 nm excitation directly on the Petri dish and band positions of all bacterial species. Average spectra of all measured strains in the susceptible, ESBL and CRE groups are shown in comparison

Figure S3C. Mean Raman spectra of UVRR and band positions of all bacterial species. Average spectra of all measured strains in the susceptible and resistant groups are shown in comparison

Figure S3C. Mean Raman spectra of UVRR and band positions of all bacterial species. Average spectra of all measured strains in the susceptible and resistant groups are shown in comparison

Figure S4. Comparison between the Raman signals of agar and bacterial colony on the Petri dish obtained with 785 nm excitation using the Raman fibre probe. The fibre's focal spot was focused on the bacterial colony, and the Raman signal was obtained. Afterwards the Petri dish was moved to blank spot and without changing focus, the Raman signal of the TSB-agar was obtained. This was done to ensure comparability of the signal intensities. 10 measurements were obtained for each bacterium and agar using 10 s integration time with 3 accumulations. This process was performed for one *E. coli* and one *K. pneumoniae* strain. Spectra were pre-processed without normalization to display the contribution of the agar signal in the bacterial spectrum. It has to be mentioned however, that the contribution of the agar signal in the Raman spectra of the bacterial colonies cannot be directly assessed and is overestimated in the Figure. In reality, the contribution of the agar-signal in the bacterial spectra is lower than shown here

Table S1. Number of strains, measured and analysed spectra per species and resistance-group for 785 nm and UVRR.

Table S2B. MIC results and resistance data for *K. pneumonie*

Table S2C. MIC results and resistance data for *K. oxitoca* and *A. baumanii*

Table S2D. MIC results and resistance data for *C. freudii* and *E. faecium*

Table S3. Raman band assignment for the UVRR.

Table S4. Raman band assignment for the Raman fibre probe with 785 nm excitation

Table S5A. Confusion matrix of training data for all machine-learning models for the classification of bacterial species

Table S5B.1. Confusion matrix of training data for all machine-learning models for the classification of *E. coli* susceptible vs resistant strains

Table S5B.2. Confusion matrix of training data for all machine-learning models for the classification of *E. coli* ESBL vs CRE

Table S5C.1. Confusion matrix of training data for all machine-learning models for the classification of *K. pneumoniae* susceptible vs resistant strains

Table S5C.2. Confusion matrix of training data for all machine-learning models for the classification of *K. pneumoniae* ESBL vs CRE

Table S5D. Confusion matrix of training data for all machine-learning models for the classification of *K. oxytoca* susceptible vs resistant strains

Table S5E.1. Confusion matrix of training data for all machine-learning models for the classification of *A. baumanii* susceptible vs resistant strains

Table S5E.2. Confusion matrix of training data for all machine-learning models for the classification of *A. baumanii* ESBL vs CRE strains

Table S5F. Confusion matrix of training data for all machine-learning models for the classification of *C. freundii* susceptible vs resistant strains

Table S5G. Confusion matrix of training data for all machine-learning models for the classification of *E. faecium* VSE vs VRE strains

Table S6. Test results of susceptible vs. resistant strains for each bacterial species

Table S7. Test results of ESBL vs. CRE strains for each bacterial species

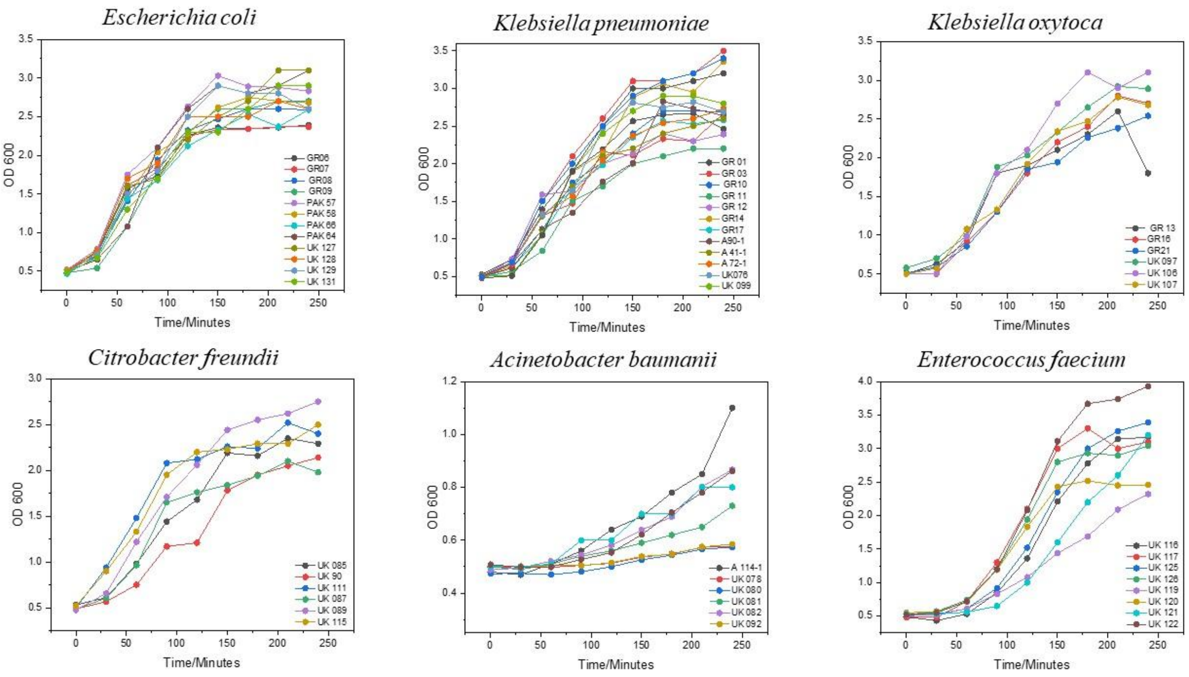

Figure S1. Grow curves of all used strains. To obtain the grow curves, a bacteria inoculum was prepared with an OD_{600} of 0.5. This was then incubated in a shaking incubator and bacteria growth was monitored by obtaining the cultures OD_{600} every 30 minutes.

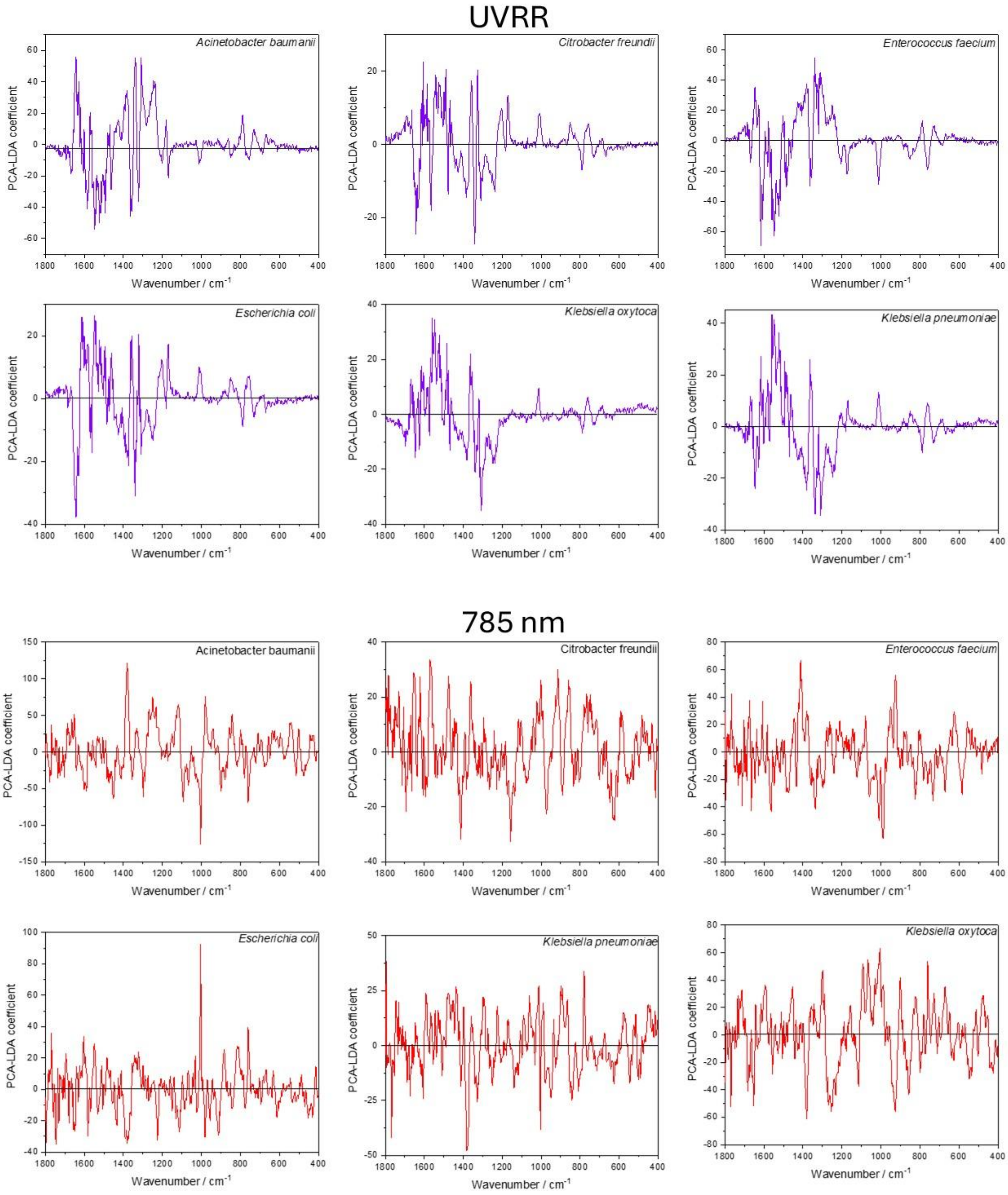

Figure S2. PCA-LDA coefficients for species classification based on Raman spectra using UVRR and 785 nm excitation. Each plot visualizes the coefficients used to identify a specific species among the others. The positive values indicate the important spectral information for each species

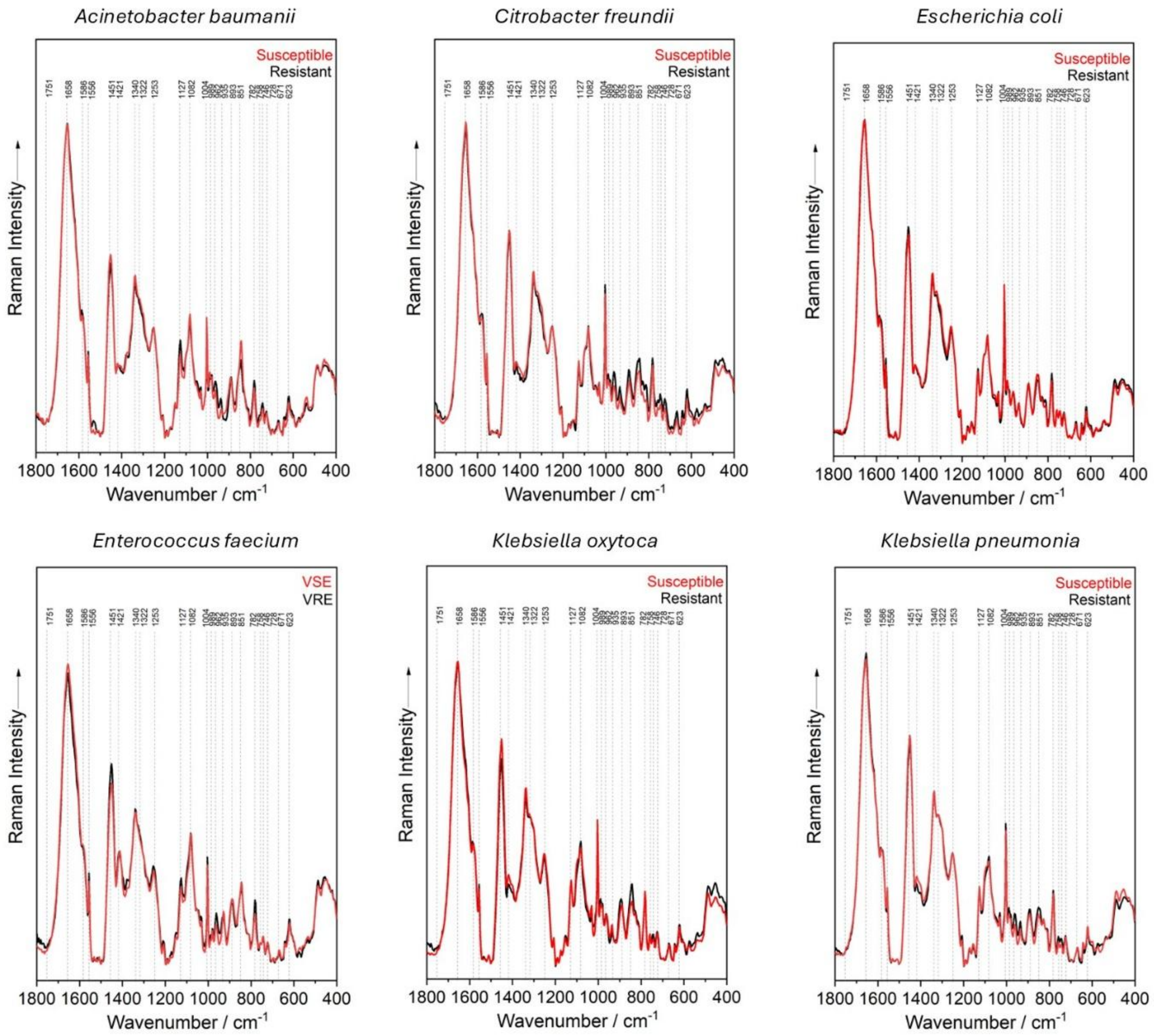

Figure S3A. Mean Raman spectra of 785 nm excitation directly on the Petri dish and band positions of all bacterial species. Average spectra of all measured strains in the susceptible and resistant groups are shown in comparison

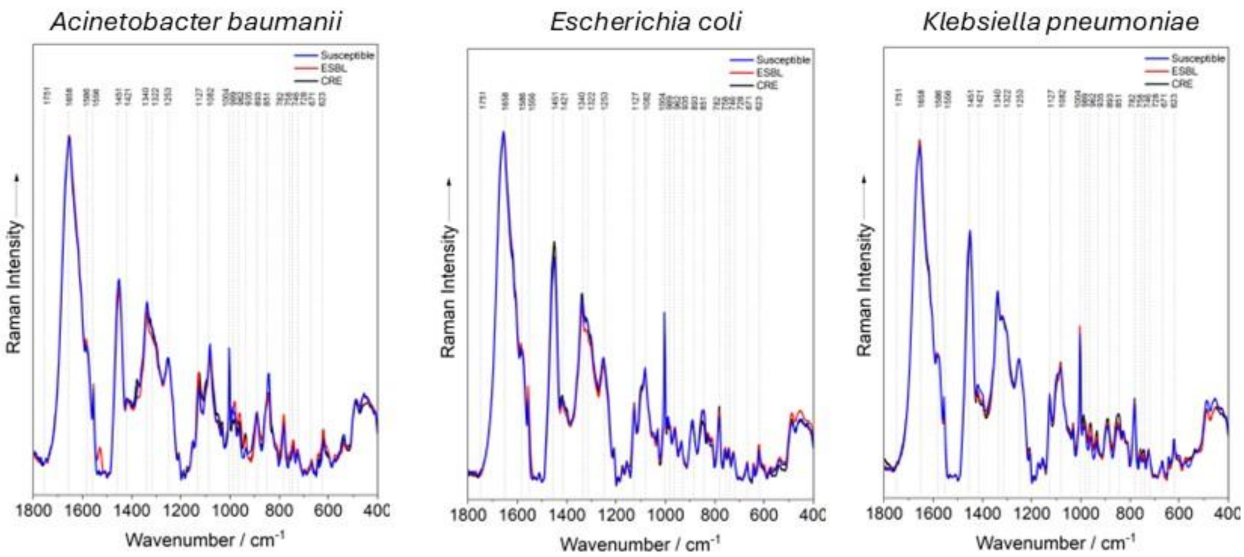

Figure S3B. Mean Raman spectra of 785 nm excitation directly on the Petri dish and band positions of all bacterial species. Average spectra of all measured strains in the susceptible, ESBL and CRE groups are shown in comparison

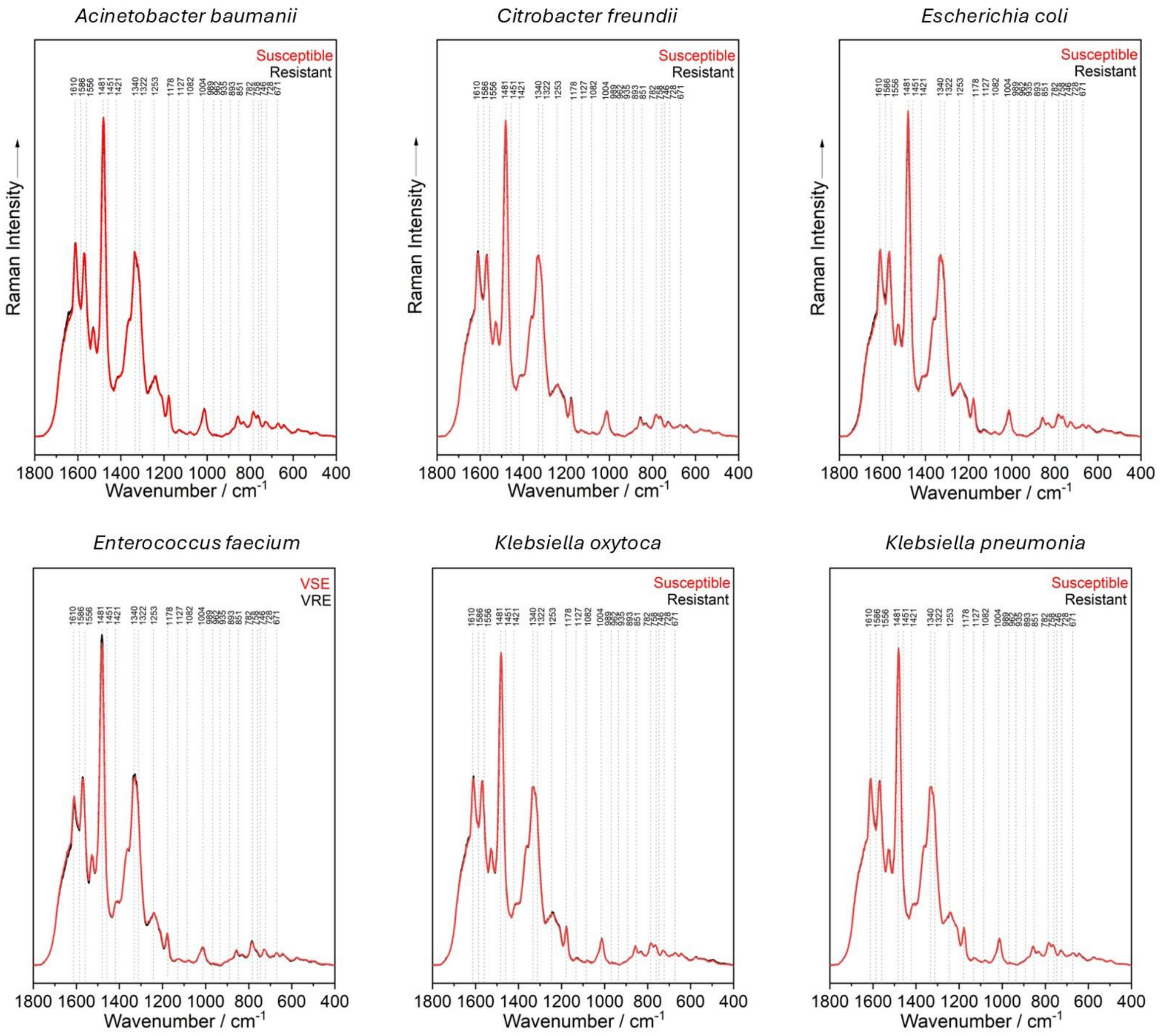

Figure S3C. Mean Raman spectra of UVRR and band positions of all bacterial species. Average spectra of all measured strains in the susceptible and resistant groups are shown in comparison

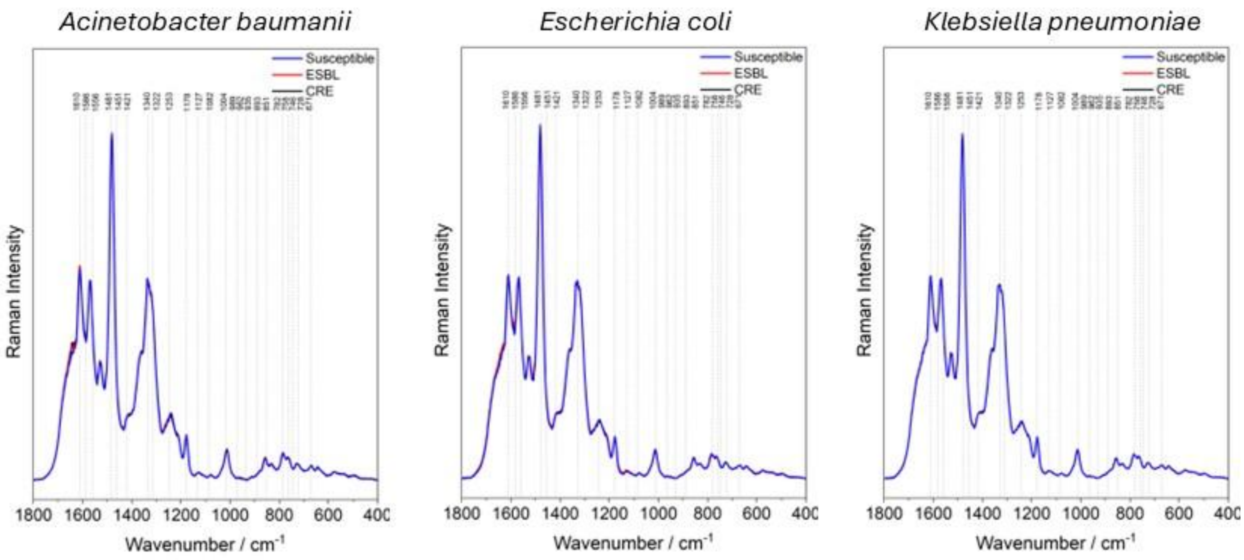

Figure S3B. Mean Raman spectra UVRR and band positions of all bacterial species. Average spectra of all measured strains in the susceptible, ESBL and CRE groups are shown in comparison.

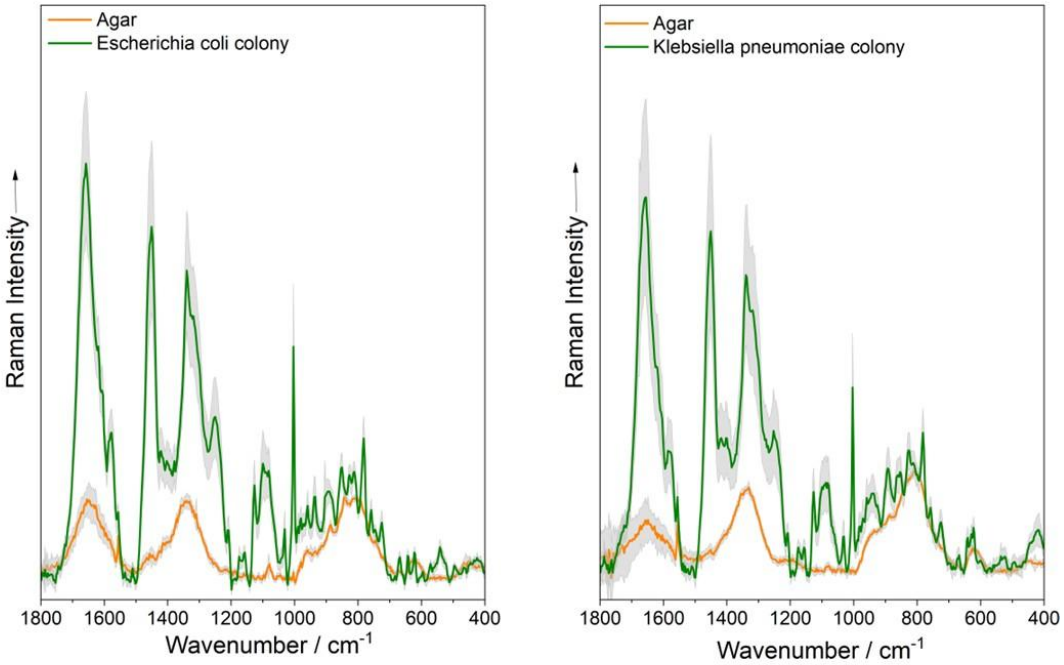

Figure S4. Comparison between the Raman signals of agar and bacterial colony on the Petri dish obtained with 785 nm excitation using the Raman fibre probe. The fibre's focal spot was focused on the bacterial colony, and the Raman signal was obtained. Afterwards the Petri dish was moved to blank spot and without changing focus, the Raman signal of the TSB-agar was obtained. This was done to ensure comparability of the signal intensities. 10 measurements were obtained for each bacterium and agar using 10 s integration time with 3 accumulations. This process was performed for one *E. coli* and one *K. pneumoniae* strain. Spectra were pre-processed without normalization to display the contribution of the agar signal in the bacterial spectrum. It has to be mentioned however, that the contribution of the agar signal in the Raman spectra of the bacterial colonies cannot be directly assessed and is overestimated in the Figure. In reality, the contribution of the agar-signal in the bacterial spectra is lower than shown here.

Table S1. Number of strains, measured and analysed spectra per species and resistance-group for 785 nm and UVRR.

	Raman fiber probe (785 nm)									
	Number of strains (number of measured spectra / number of analysed spectra)									
	Susceptible		ESBL		CRE		VSE		VRE	
	CV	Test	CV	Test	CV	Test	CV	Test	CV	Test
Escherichia coli	4 (258/240)	1 (21/21)	4 (254/240)	1 (22/22)	4 (255/218)	1 (22/22)				
Klebsiella pneumoniae	5 (315/313)	1 (22/22)	5 (299/289)	1 (22/22)	5 (317/316)	1 (23/23)				
Klebsiella oxytoca	3 (191/187)	1 (22/22)	---	---	3 (187/166)	1 (22/22)				
Acinetobacter baumanii	3 (196/151)	1 (22/21)	3 (192/169)	1 (21/21)	3 (192/172)	1 (21/19)				
Citrobacter freundii	3 (195/169)	1 (22/22)	3 (167/128)	1 (21/19)	---	---				
Enterococcus faecium							4 (245/195)	1 (22/16)	4 (249/196)	1 (21/12)
	UVRR (244 nm)									
	Number of strains (number of measured spectra / number of analysed spectra)									
	Susceptible		ESBL		CRE		VSE		VRE	
	CV	Test	CV	Test	CV	Test	CV	Test	CV	Test
Escherichia coli	4 (1198/1175)	1 (100/89)	4 (1166/1087)	1 (100/97)	4 (1247/1114)	1 (99/95)				
Klebsiella pneumoniae	5 (1494/1440)	1 (100/65)	5 (1494/1439)	1 (99/89)	5 (1460/1369)	1 (100/95)				
Klebsiella oxytoca	3 (898/868)	1 (100/91)	---	---	3 (796/783)	1 (100/64)				
Acinetobacter baumanii	3 (872/782)	1 (100/89)	3 (898/761)	1 (100/54)	3 (899/743)	1 (100/99)				
Citrobacter freundii	3 (900/855)	1 (100/100)	3 (895/806)	1 (100/100)	---	---				
Enterococcus faecium							4 (1199/1086)	1 (100/98)	4 (1234/1156)	1 (100/100)

Table S2A. MIC results and resistance data for *E. coli*

	Escherichia coli											
	Susceptible				ESBL				CRE			
	UK128	UK129	UK131	UK144	Gr06	Gr07	Gr08	Gr09	PAK57	PAK58	PAK64	PAK66
MIC Amikacin					8	<=4	<=2	16	<=2	4	<=2	8
Amikacin					I	S	S	I	S	I	S	I
MIC Amoxicillin/ Clavulanic Acid					4	8/2	16	>=32				
Amoxicillin/ Clavulanic Acid					S	S	R	R				
MIC Ampicillin	<=2	>=32	<=2	8	>=32	>8	>=32	>=32				
Ampicillin	S	R	S	S	R	R	R	R				
MIC Ampicillin/ Sulbactam	<=2	>=32	<=2	<=2	16		>=32	>=32				
Ampicillin/ Sulbactam	S	R	S	S	R		R	R				
MIC Aztreonam					16		16	16	>=64	>=64	<=1	>=64
Aztreonam					R		R	R	R	R	I	R
MIC Cefepime					2	4	2	2	>=64	>=64	8	>=64
Cefepime					I	I	I	I	R	R	R	R
MIC Cefixime					>=4		>=4	>=4				
Cefixime					R		R	R				
MIC Cefotaxime	<=0,25	<=0,25	<=0,25	<=0,25	32		>=64	8	>=64	>=64	>=64	>=64
Cefotaxime	S	S	S	S	R		R	R	R	R	R	R
MIC Ceftazidime	<=0,25	0,25	0,25	<=0,12	4	8	16	>=64	>=64	>=64	>=64	>=64
Ceftazidime	S	S	S	S	I	R	R	R	R	R	R	R
MIC Ceftriaxone					>=64	>4	>=64	16				
Ceftriaxone					R	R	R	R				
MIC Cefuroxime	2	4	4		>=64	>8	>=64	>=64				
Cefuroxime	I	I	I	4	R	R	R	R				
MIC Cefpodoxim	<=0,25	<=0,25	0,5	I								
Cefpodoxim	S	S	S	0,5								
MIC Cefuroxim Axetil	2	4	4	S	>=64		>=64	>=64				
Cefuroxim Axetil	S	S	S	4	R		R	R				
MIC Chloramphenicol				S	8		8	8				
Chloramphenicol					S		S	S				
MIC Ciprofloxacin	<=0,06	>=4	<=0,06	1	<=0,25	>1	>=4	>=4	>=4	>=4	>=4	>=4
Ciprofloxacin	S	R	S	R	S	R	R	R	R	R	R	R
MIC Ertapenem					<=0,5	<=0,25	<=0,5	<=0,5				
Ertapenem					S	S	S	S				
MIC Gentamicin	<=1	<=1	<=1		<=1	2	>=16	<=1	<=1	>=16	<=1	<=1
Gentamicin	S	S	S	<=1	S	S	R	S	S	R	S	S
High level resistance to Gentamicin				S								
MIC Imipenem	<=0,25	<=0,25	<=0,25	<=0,25	<=0,25	<=0,25	<=0,25	<=0,25	8	>=16	>=16	>=16
Imipenem	S	S	S	S	S	S	S	S	I	R	R	R
MIC Levofloxacin					<=0,12		>=8	>=8				
Levofloxacin					S		R	R				
MIC Meropenem	<=0,25	<=0,25	<=0,25	<=0,25	<=0,25	<=0,125	<=0,25	<=0,25	>=16	>=16	>=16	8
Meropenem	S	S	S	S	S	S	S	S	R	R	R	I
MIC Linezolid												
Linezolid												
MIC Moxifloxacin					<=0,25		>=8	>=8	>=8	>=8	>=8	>=8
Moxifloxacin					S		R	R	R	R	R	R
MIC Nitrofurantoin	<=16	32	32	<=16	<=16	<=16	128	<=16				
Nitrofurantoin	S	S	S	S	S	S	R	S				
MIC Ofloxacin					<=0,25		>=8	>=8				
Ofloxacin					S		R	R				
MIC Piperacillin					>=128		>=128	>=128	>=128	>=128	>=128	>=128
Piperacillin					R		R	R	R	R	R	R
MIC Piperacillin/ Tazobactam	<=4	<=4	<=4	<=4	<=4	<=4/4	8	64	>=128	>=128	>=128	>=128
Piperacillin/ Tazobactam	S	S	S	S	S	S	S	R	R	R	R	R
MIC Ticarcillin					>=128		>=128	>=128				
MIC Quinupristin/Dalfopristin												
Quinupristin/Dalfopristin												
Ticarcillin					R		R	R				
MIC Teicoplanin												
Teicoplanin												
MIC Ticarcillin/ Clavulanic Acid					<=8		16	>=128				
Ticarcillin/ Clavulanic Acid					S		I	R				
MIC Tigecycline	<=0,5	<=0,5	<=0,5		<=0,5		<=0,5	<=0,5	<=0,5	<=0,5	<=0,5	<=0,5
Tigecycline	S	S	S	<=0,5	S		S	S	S	S	S	S
MIC Tobramycin				S	>=16		>=16	>=16	<=1	>=16	<=1	>=16
Tobramycin					R		R	R	S	R	S	R
MIC Trimethoprim/ Sulfamethoxazole	<=20	>=320	<=20	<=20	>=320	>4/76	<=20	<=20	>=320	>=320	<=20	>=320
Trimethoprim/ Sulfamethoxazole	S	R	S	S	R	R	S	S	R	R	S	R
MIC Fosfomycin									<=16	<=16	<=16	<=16
Fosfomycin									S	S	S	S
MIC Vancomycin												
Vancomycin												
MIC Colisin									<=0,5	<=0,5	<=0,5	<=0,5
Colistin									S	S	S	S

Table S2B. MIC results and resistance data for *K. pneumonie*

	Klebsiella pneumoniae														
	Susceptible					ESBL					CRE				
	UK076	UK099	UK102	UK104	UK110	Gr01	Gr03	Gr10	Gr11	Gr12	Gr14	Gr17	A90-1	A41-1	A72-1
MIC Amikacin						<=4	<=4	16	<=4	>16	>=64	>=64	>=64	>=64	>=64
Amikacin						S	S	I	I	R	R	R	R	R	R
MIC Amoxicillin/ Clavulanic Acid						8/2	>32/2	>32/2	>32/2	>32/2	>=32	>=32			
Amoxicillin/ Clavulanic Acid						S	R	R	R	R	R	R			
MIC Ampicillin	16	16	>=32	>=32	16	>8	>8	>8	>8	>8	>=32	>=32			
Ampicillin	R	R	R	R	R	R	R	R	R	R	R	R			
MIC Ampicillin/ Sulbactam	4	<=2	16	4	<=2						>=32	>=32			
Ampicillin/ Sulbactam	S	S	R	S	R						R	R			
MIC Aztreonam											>=64	>=64	>=64	>=64	>=64
Aztreonam											R	R	R	R	R
MIC Cefepime						<=1	8	>8	>8	>8	>=64	>=64	>=64	>=64	>=64
Cefepime						S	R	R	R	R	R	R	R	R	R
MIC Cefixime											>=4	>=4			
Cefixime											R	R			
MIC Cefotaxime	<=0.25	<=0.25	<=0.25	<=0.25	<=0.25	0,5					>=64	>=64	>=64	>=64	>=64
Cefotaxime	S	S	S	S	S	S					R	R	R	R	R
MIC Ceftazidime	<=0.12	<=0.12	<=0.12	<=0.12	<=0.12	1	>8	>8	>8	>8	32	>=64	>=64	>=64	>=64
Ceftazidime	S	S	S	S	S	S	R	R	R	R	R	R	R	R	R
MIC Ceftriaxone							>4	>4	>4	>4	>=64	>=64			
Ceftriaxone							R	R	R	R	R	R			
MIC Cefuroxime	<=1	<=1	<=1	2	<=1	>8	>8	>8	>8	>8	>=64	>=64			
Cefuroxime	I	I	I	I	I	R	R	R	R	R	R	R			
MIC Cefpodoxim	<=0.25	<=0.25	<=0.25	<=0.25	<=0.25										
Cefpodoxim	S	S	S	S	S										
MIC Cefuroxim Axetil	<=1	<=1	<=1	2	<=1						>=64	>=64			
Cefuroxim Axetil	S	S	S	S	I						R	R			
MIC Chloramphenicol											16	>=64			
Chloramphenicol											R	R			
MIC Ciprofloxacin	<=0.06	<=0.06	<=0.06	<=0.06	<=0.06	<=0,25	>1	>1	>1	>1	>=4	>=4	>=4	>=4	>=4
Ciprofloxacin	S	S	S	S	S	S	R	R	R	R	R	R	R	R	R
MIC Ertapenem						<=0,25	<=0,25	0,5	>1	>1	>=8	>=8			
Ertapenem						S	S	S	R	R	R	R			
MIC Gentamicin	>=1	<=1	<=1	<=1	<=1	<=1	<=1	2	2	4	>=16	>=16	>=16	>=16	>=16
Gentamicin	S	S	S	S	S	S	S	S	S	I	R	R	R	R	R
High level resistance to Gentamicin															
MIC Imipenem	<=0.25	<=0.25	<=0.25	<=0.25	<=0.25	<=0,25	0,5	<=0,25	8	>8	>=16	>=16	>=16	8	>=16
Imipenem	S	S	S	S	S	S	S	S	R	R	R	R	R	I	R
MIC Levofloxacin						1			>2	>2	>=8	>=8			
Levofloxacin						S			R	R	R	R			
MIC Meropenem	<=0.25	<=0.25	<=0.25	<=0.25	<=0.25	<=0,125		<=0,125	>8	>8	>=16	>=16	>=16	>=16	>=16
Meropenem	S	S	S	S	S	S		S	R	R	R	R	R	R	R
MIC Linezolid															
Linezolid															
MIC Moxifloxacin											>=8	>=8	>=8	>=8	>=8
Moxifloxacin											R	R	R	R	R
MIC Nitrofurantoin															
Nitrofurantoin															
MIC Ofloxacin										2	>=8				
Ofloxacin										R	R				
MIC Piperacillin						16			>16	>=128	>=128		>=128	>=128	>=128
Piperacillin						I			R	R	R		R	R	R
MIC Piperacillin/ Tazobactam	<=4	<=4	<=4	<=4	<=4	16/4	>16/4	16/4	>16/4	>=128	>=128	>16/4	>=128	>=128	>=128
Piperacillin/ Tazobactam	S	S	S	S	S	I	R	I	R	R	R	R	R	R	R
MIC Ticarcillin										>=128	>=128				
MIC Quinupristin/Dalfopristin															
Quinupristin/Dalfopristin															
Ticarcillin										R	R				
MIC Teicoplanin															
Teicoplanin															
MIC Ticarcillin/ Clavulanic Acid										>=128	>=128				
Ticarcillin/ Clavulanic Acid										R	R				
MIC Tigecycline						6				>=16			2	>=8	>=8
Tigecycline						R				R			I	R	R
MIC Tobramycin						<=1			>4	>=320	8		>=16	>=16	>=16
Tobramycin						S			R	R	R		R	R	R
MIC Trimethoprim/ Sulfamethoxazole	<=20	<=20	<=20	<=20	<=20	<=1/19	>4/76	>4/76	>4/76	<=1/19	>=320	>4/76	>=320	>=320	>=320
Trimethoprim/ Sulfamethoxazole	S	S	S	S	S	S	R	R	R	S	R	R	R	R	R
MIC Fosfomycin	<=16	<=16	<=16	<=16	<=16								64	64	64
Fosfomycin	S	S	S	S	S								R	R	R
MIC Vancomycin															
Vancomycin															
MIC Colisin													<=0,5	>=16	<=0,5
Colistin													S	R	S

Table S2C. MIC results and resistance data for *K. oxitoca* and *A. baumanii*

	Klebsiella oxytoca						*Acinetobacter baumanii*								
	Susceptible			CRE			Susceptible			ESBL			CRE		
	UK097	UK106	UK107	Gr13	Gr16	Gr21	UK092	UK094	UK096	UK080	UK081	UK082	A90-2	A114-1	UK078
MIC Amikacin				16	16	16							>=64	>=64	
Amikacin				I	I	I							R	R	
MIC Amoxicillin/ Clavulanic Acid				>=32	>=32	>=32									
Amoxicillin/ Clavulanic Acid				R	R	R									
MIC Ampicillin	>=32	4	>=32	>=32	>=32	>=32									
Ampicillin	R	R	R	R	R	R									
MIC Ampicillin/ Sulbactam	8	<=2	>=32	>=32	>=32	>=32									
Ampicillin/ Sulbactam	S	R	R	R	R	R									
MIC Aztreonam				>=64	>=64	>=64							>=64	>=64	
Aztreonam				R	R	R							R	R	
MIC Cefepime				8	8	8							>=64	>=64	
Cefepime				R	R	R							R	R	
MIC Cefixime				>=4	>=4	>=4									
Cefixime				R	R	R									
MIC Cefotaxime	<=0.25	<=0.25	<=0.25	>=64	>=64	>=64							>=64	>=64	
Cefotaxime	S	S	S	R	R	R							R	R	
MIC Ceftazidime	<=0.12	<=0.12	<=0.12	>=64	>=64	>=64							>=64	>=64	
Ceftazidime	S	S	S	R	R	R							R	R	
MIC Ceftriaxone				>=64	16	16									
Ceftriaxone				R	R	R									
MIC Cefuroxime	<=1	<=1	4	>=64	>=64	>=64									
Cefuroxime	I	I	I	R	R	R									
MIC Cefpodoxim	<=0.25	<=0.25	<=0.25												
Cefpodoxim	S	S	S												
MIC Cefuroxim Axetil	<=1	<=1	4	>=64	>=64	>=64									
Cefuroxim Axetil	S	S	S	R	R	R									
MIC Chloramphenicol				>=64	>=64	>=64									
Chloramphenicol				R	R	R									
MIC Ciprofloxacin	<=0.06	<=0.06	<=0.06	1	1	1	0,5	0,5	0,12	0,12	0,25	0,12	>=4	>=4	>=4
Ciprofloxacin	S	S	S	R	R	R	I	I	S	I	I	I	R	R	R
MIC Ertapenem				4	4	4									
Ertapenem				R	R	R									
MIC Gentamicin	<=1	<=1	<=1	>=16	>=16	>=16	<=1	<=1	<=1	<=1	<=1	<=1	>=16	>=16	>=16
Gentamicin	S	S	S	R	R	R	S	S	S	S	S	S	R	R	R
High level resistance to Gentamicin															
MIC Imipenem	<=0.25	<=0.25	<=0.25	8	8	>=16	<=0.5	0,5	<=0.5	<=0.5	<=0.5	<=0.5	>=16	>=16	>=16
Imipenem	S	S	S	R	R	R	S	S	S	S	S	S	R	R	R
MIC Levofloxacin				1	1	1									
Levofloxacin				I	I	I									
MIC Meropenem	<=0.25	<=0.25	<=0.25	>=16	>=16	>=16	<=0.25	<=0.25	0,5	<=0.25	<=0.25	<=0.25	>=16	>=16	>=16
Meropenem	S	S	S	R	R	R	S	S	S	S	S	S	R	R	R
MIC Linezolid															
Linezolid															
MIC Moxifloxacin				4	4	2									
Moxifloxacin				R	R	R									
MIC Nitrofurantoin															
Nitrofurantoin															
MIC Ofloxacin				>=8	>=8	>=8									
Ofloxacin				R	R	R									
MIC Piperacillin				>=128	>=128	>=128							>=128	>=128	
Piperacillin				R	R	R							R	R	
MIC Piperacillin/ Tazobactam	<=4	<=4	<=4	>=128	>=128	>=128							>=128	>=128	
Piperacillin/ Tazobactam	S	S	S	R	R	R							R	R	
MIC Ticarcillin				>=128	>=128	>=128									
MIC Quinupristin/Dalfopristin															
Quinupristin/Dalfopristin															
Ticarcillin				R	R	R									
MIC Teicoplanin															
Teicoplanin															
MIC Ticarcillin/ Clavulanic Acid				>=128	>=128	>=128									
Ticarcillin/ Clavulanic Acid				R	R	R									
MIC Tigecycline													2	2	
Tigecycline													I	I	
MIC Tobramycin				>=16	>=16	>=16							>=16	>=16	
Tobramycin				R	R	R							R	R	
MIC Trimethoprim/ Sulfamethoxazole	<=20	<=20	<=20	>=320	>=320	<=20	<=20	<=20	<=20	<=20	<=20	<=20	<=20	>=320	>=320
Trimethoprim/ Sulfamethoxazole	S	S	S	R	R	S	S	S	S	S	S	S	S	R	R
MIC Fosfomycin	64	<=16	32												
Fosfomycin	R	S	S												
MIC Vancomycin															
Vancomycin															
MIC Colisin													8	<=0,5	
Colistin													R	S	

Table S2D. MIC results and resistance data for *C. freudii* and *E. faecium*

	Citrobacter freundii						*Enterococcus faecium*							
	Susceptible			ESBL			VSE				VRE			
	UK115	UK087	UK089	UK085	UK090	UK111	UK119	UK120	UK121	UK122	UK125	UK126	UK116	UK117
MIC Amikacin														
Amikacin														
MIC Amoxicillin/ Clavulanic Acid														
Amoxicillin/ Clavulanic Acid														
MIC Ampicillin	4	8	>=32	>=32	16	>=32	>=32	>=32	>=32	>=32	>=32	>=32	>=32	>=32
Ampicillin	R	R	R	R	R	R	R	R	R	R	R	R	R	R
MIC Ampicillin/ Sulbactam	<=2	<=2	16	>=32	>=32	>=32	>=32	>=32	>=32	>=32	>=32	>=32	>=32	>=32
Ampicillin/ Sulbactam	R	R	R	R	R	R	R	R	R	R	R	R	R	R
MIC Aztreonam														
Aztreonam														
MIC Cefepime														
Cefepime														
MIC Cefixime														
Cefixime														
MIC Cefotaxime	<=0.25	<=0.25	0,5	32	4	32								
Cefotaxime	S	S	S	R	R	R								
MIC Ceftazidime	0,25	0,25	0,5	32	0,5	>=64								
Ceftazidime	S	S	S	R	R	R								
MIC Ceftriaxone														
Ceftriaxone														
MIC Cefuroxime														
Cefuroxime														
MIC Cefpodoxim	2	2	2	>=8	4	>=8								
Cefpodoxim	R	R	R	R	R	R								
MIC Cefuroxim Axetil														
Cefuroxim Axetil														
MIC Chloramphenicol														
Chloramphenicol														
MIC Ciprofloxacin	0,25	<=0.06	<=0.06	>=4	>=4	<=0.06								
Ciprofloxacin	S	S	S	R	R	S								
MIC Ertapenem														
Ertapenem														
MIC Gentamicin	<=1	<=1	<=1	>=16	<=1	<=1								
Gentamicin	S	S	S	R	S	S								
High level resistance to Gentamicin							neg	neg	neg	neg	neg	neg	neg	neg
MIC Imipenem	1	1	<=0.25	0,5	1	1	>=16	>=16	>=16	>=16	>=16	>=16	>=16	>=16
Imipenem	S	S	S	S	S	S	R	R	R	R	R	R	R	R
MIC Levofloxacin							>=8	>=8	>=8	>=8	>=8	>=8	>=8	>=8
Levofloxacin							R	R	R	R	R	R	R	R
MIC Meropenem	<=0.25	<=0.25	<=0.25	<=0.25	<=0.25	<=0.25								
Meropenem	S	S	S	S	S	S								
MIC Linezolid							4	2	2	2	2	2	2	1
Linezolid							S	S	S	S	S	S	S	S
MIC Moxifloxacin														
Moxifloxacin														
MIC Nitrofurantoin														
Nitrofurantoin														
MIC Ofloxacin														
Ofloxacin														
MIC Piperacillin														
Piperacillin														
MIC Piperacillin/ Tazobactam	<=4	<=4	<=4	>=128	<=4	64								
Piperacillin/ Tazobactam	S	S	S	R	I	R								
MIC Ticarcillin														
MIC Quinupristin/Dalfopristin							2	0,5	0,5	0,5	0,5	0,5	0,5	0,5
Quinupristin/Dalfopristin							I	S	S	S	S	S	S	S
Ticarcillin														
MIC Teicoplanin							<=0.5	<=0.5	<=0.5	<=0.5	>=32	>=32	<=0.5	<=0.5
Teicoplanin							S	S	S	S	R	R	S	S
MIC Ticarcillin/ Clavulanic Acid														
Ticarcillin/ Clavulanic Acid														
MIC Tigecycline							<=0.12	<=0.12	<=0.12	<=0.12	<=0.12	0,25	<=0.12	<=0.12
Tigecycline							S	S	S	S	S	S	S	S
MIC Tobramycin														
Tobramycin														
MIC Trimethoprim/ Sulfamethoxazole	<=20	<=20	<=20	>=320	<=20	<=20								
Trimethoprim/ Sulfamethoxazole	S	S	S	R	S	S								
MIC Fosfomycin	<=16	<=16	32	<=16	<=16	<=16								
Fosfomycin	S	S	R	S	S	S								
MIC Vancomycin							<=0.5	<=0.5	<=0.5	<=0.5	>=32	>=32	>=32	>=32
Vancomycin							S	S	S	S	R	R	R	R
MIC Colisin														
Colistin														

Table S3. Raman band assignment for the UVRR.

Wavenumber / cm^{-1}	Assignment	Biomolecule	Reference
1610	ν(C=C) ring vibrations of phenylalanine, tyrosine (1606)	Protein	1
1568	ν (ring) Guanine, Adenine (1577)	Nucleic acids	2
1529	Cytosine (1529)	Nucleic acids	3
1481	Stretch along the long axis of the purines (Guanine, Adenine) (1481)	Nucleic acids	2
1412			
1361	Cytosine (1365), thymine (1369)	Nucleic acids	3-5
1328	Adenine, Guanine, Tyrosine (1330)	Nucleic acids, Proteins	3
1238	Guanine, Adenine, Uracile (1230)	Nucleic acids	3
1178	C-C and C-N stretching vibration of Cytosine and Thymine (1175), Tyrosine in-plane C-H bending vibration (1175)	Nucleic acids, Proteins	4, 6
1013	Phenylalanine ring breathing (1006)	Proteins	1, 7
857	Ring breathing vibration of Tyrosine (854)	Proteins	1, 8
830	Ring breathing vibration of Tyrosine (829)	Protein	4
785	Ring breathing modes of cytosine, uracil and thymine (780-786)	Nucleic acids	1
764	Ring breathing vibration of Tryptophan (760)	Proteins	9
728	Ring breathing modes of adenine (720-730)	Nucleic acids	3, 8, 10

Table S4. Raman band assignment for the Raman fibre probe with 785 nm excitation.

Wavenumber / cm^{-1}	Assignment	Biomolecule	Reference
1751	C=O ester stretching vibration (1727-1749)	Lipids	6, 7
1658	Amide I (1650-1680), C=C stretching vibration of lipids (1653-1672)	Protein, Lipids	6, 11
1586	N-H bending and C-N stretching vibration of guanine and cytosine (1594)	Nucleic acids	12
1556	Tryptophan: C-C stretching vibration of the pyrrole ring (1551)	Protein	4
1451	CH_2/CH_3 deformation of lipids (1433-1468) and proteins (1431-1481) and carbohydrates	Lipids, Proteins, carbohydrates	6, 13, 14
1421	CH_2 deformation vibration (1419-1428)	Lipids	6
1340	CH_2 deformation vibration of proteins (1310-1348), ring vibrations of guanine and adenine (1337	Nucleic acids, protein	1, 14
1322	Guanine (1319), CH_2 deformation vibration of proteins (1310-1348), CH_2/CH_3 twist of lipids (1319)	Nucleic acids, protein, lipids	1, 14
1253	=C-H deformation vibration (1249-1287), Amide III (1240–1265)	Protein, lipids	1, 6
1127	C–C stretching (~1130), DNA backbone PO_2 symmetric stretching vibration	Lipids, Nucleic acids	10
1082	PO_2 symmetric stretching vibration of nucleic acids (1095-1060), C-N stretching vibration of proteins (1095-1060), C-C and C-O stretching vibration of lipids (1095-1060)	Nucleic acids, Protein, Lipids	9
1004	Phenylalanine ring breathing vibration (1006)	Protein	1, 7
962	Tyrosine (960)	Protein	1
935	DNA backbone stretching vibration (929), C-C stretching vibration of proteins (929)	Nucleic acids, Proteins	15
893	C–O–C glycosidic ring stretching vibration (897), (C-O-O) skeletal vibration of lipids (866-898)	Carbohydrates, lipids	6, 7
851	Ring breathing vibration of Tyrosine (854)	Protein	1, 8
782	Ring breathing modes of cytosine, uracil and thymine (780-786), O—P—O stretching DNA backbone (788)	Nucleic acids	1, 10
758	Ring breathing vibration of Tryptophan (760)	Protein	9
746	Ring breathing vibration of thymine (748)	Protein	1, 11
728	Ring breathing modes of adenine (720-730)	Nucleic acids	3, 8, 10
623	Phenylalanine C-C twisting vibration (620)	Protein	1, 8

Table S5A. Confusion matrix and 95% confidence intervals (CI) of sensitivity of training data for all machine-learning models for the classification of bacterial species

		TRUE	PREDICTED								
			A. baumanii	*C. freundii*	*E. faecium*	*E. coli*	*K. oxytoca*	*K. pneumoniae*	Sensitivity	Specificity	Sensitivity (95% CI)
**PCA-LDA**	**785 nm**	*A. baumanii*	8	1	0	0	0	0	0.889	1	0.59–0.99
		C. freundii	0	3	0	1	0	2	0.5	0.98	0.17–0.83
		E. faecium	0	0	6	0	0	2	0.75	1	0.41–0.94
		E. coli	0	0	0	12	0	0	1	0.977	0.81–1.00
		K. oxytoca	0	0	0	0	5	1	0.833	0.98	0.44–0.98
		K. pneumoniae	0	0	0	0	1	14	0.933	0.878	0.73–0.99
	**UVRR**	*A. baumanii*	9	0	0	0	0	0	1	1	0.76–1.00
		C. freundii	0	5	0	0	1	0	0.833	0.98	0.44–0.98
		E. faecium	0	0	8	0	0	0	1	1	0.74–1.00
		E. coli	0	1	0	11	0	0	0.917	0.977	0.67–0.99
		K. oxytoca	0	0	0	0	4	2	0.667	0.84	0.29–0.92
		K. pneumoniae	0	0	0	1	7	7	0.467	0.951	0.24–0.71
**PCA-SVM**	**785 nm**	*A. baumanii*	7	1	1	0	0	0	0.778	0.979	0.46–0.95
		C. freundii	1	3	0	0	0	2	0.5	0.96	0.17–0.83
		E. faecium	0	0	6	1	0	1	0.75	0.938	0.41–0.94
		E. coli	0	0	0	11	0	1	0.917	0.955	0.67–0.99
		K. oxytoca	0	1	0	0	4	1	0.667	0.98	0.29–0.92
		K. pneumoniae	0	0	2	1	1	11	0.733	0.878	0.48–0.90
	**UVRR**	*A. baumanii*	9	0	0	0	0	0	1	1	0.76–1.00
		C. freundii	0	5	0	0	1	0	0.833	0.981	0.44–0.98
		E. faecium	0	0	8	0	0	0	1	1	0.74–1.00
		E. coli	0	1	0	11	0	0	0.917	0.955	0.67–0.99
		K. oxytoca	0	0	0	0	3	3	0.5	0.92	0.17–0.83
		K. pneumoniae	0	0	0	2	3	10	0.667	0.927	0.42–0.86
**PLS-DA**	**785 nm**	*A. baumanii*	8	1	0	0	0	0	0.889	1	0.59–0.99
		C. freundii	0	6	0	0	0	0	1	0.94	0.67–1.00
		E. faecium	0	0	7	1	0	0	0.875	0.979	0.55–0.99
		E. coli	0	0	0	12	0	0	1	0.955	0.81–1.00
		K. oxytoca	0	0	0	0	6	0	1	0.94	0.67–1.00
		K. pneumoniae	0	2	1	1	3	8	0.533	1	0.29–0.76
	**UVRR**	*A. baumanii*	9	0	0	0	0	0	1	1	0.76–1.00
		C. freundii	0	5	0	1	0	0	0.833	0.94	0.44–0.98
		E. faecium	0	0	8	0	0	0	1	1	0.74–1.00
		E. coli	0	2	0	10	0	0	0.833	0.977	0.56–0.96
		K. oxytoca	0	1	0	0	4	1	0.667	0.78	0.29–0.92
		K. pneumoniae	0	0	0	0	11	4	0.267	0.976	0.10–0.52

Table S5A continue. Confusion matrix and 95% confidence intervals (CI) of sensitivity of training data for all machine-learning models for the classification of bacterial species

		TRUE	PREDICTED								
			A. baumanii	*C. freundii*	*E. faecium*	*E. coli*	*K. oxytoca*	*K. pneumoniae*	Sensitivity	Specificity	Sensitivity (95% CI)
PCA-RF	***785 nm***	*A. baumanii*	8	1	0	0	0	0	0.889	0.979	0.59–0.99
		C. freundii	1	1	0	2	0	2	0.167	0.98	0.02–0.56
		E. faecium	0	0	7	0	0	1	0.875	1	0.55–0.99
		E. coli	0	0	0	11	0	1	0.917	0.955	0.67–0.99
		K. oxytoca	0	0	0	0	3	3	0.5	1	0.17–0.83
		K. pneumoniae	0	0	0	0	0	15	1	0.829	0.85–1.00
	UVRR	*A. baumanii*	9	0	0	0	0	0	1	1	0.76–1.00
		C. freundii	0	1	0	4	0	1	0.167	1	0.02–0.56
		E. faecium	0	0	8	0	0	0	1	1	0.74–1.00
		E. coli	0	0	0	4	0	8	0.333	0.864	0.12–0.61
		K. oxytoca	0	0	0	0	0	6	0	1	0.00–0.33
		K. pneumoniae	0	0	0	2	0	13	0.867	0.634	0.64–0.97

Table S5B.1. Confusion matrix and 95% confidence intervals (CI) of sensitivity of training data for all machine-learning models for the classification of *E. coli* susceptible vs resistant strains

		TRUE	PREDICTED				
			Resistant	Susceptible	Sensitivity	Specificity	Sensitivity (95% CI)
**PCA-LDA**	**785 nm**	Resistant	7	1	0.875	1	0.55–0.99
		Susceptible	0	4	1	0.875	0.56–1.00
	**UVRR**	Resistant	8	0	1	1	0.74–1.00
		Susceptible	0	4	1	1	0.56–1.00
**PCA-SVM**	**785 nm**	Resistant	8	0	1	0.5	0.74–1.00
		Susceptible	2	2	0.5	1	0.12–0.88
	**UVRR**	Resistant	8	0	1	1	0.74–1.00
		Susceptible	0	4	1	1	0.56–1.00
**PLS-DA**	**785 nm**	Resistant	6	2	0.75	1	0.41–0.94
		Susceptible	0	4	1	0.75	0.56–1.00
	**UVRR**	Resistant	8	0	1	1	0.74–1.00
		Susceptible	0	4	1	1	0.56–1.00
**PCA-RF**	**785 nm**	Resistant	8	0	1	1	0.74–1.00
		Susceptible	0	4	1	1	0.56–1.00
	**UVRR**	Resistant	8	0	1	0.5	0.74–1.00
		Susceptible	2	2	0.5	1	0.12–0.88

Table S5B.2. Confusion matrix and 95% confidence intervals (CI) of sensitivity of training data for all machine-learning models for the classification of *E. coli* ESBL vs CRE

		TRUE	PREDICTED				
			CRE	ESBL	Sensitivity	Specificity	Sensitivity (95% CI)
PCA-LDA	785 nm	CRE	3	1	0.75	0.5	0.28–0.97
		ESBL	2	2	0.5	0.75	0.12–0.88
	UVRR	CRE	3	1	0.75	0	0.28–0.97
		ESBL	4	0	0	0.75	0.00–0.44
PCA-SVM	785 nm	CRE	2	2	0.5	0.5	0.12–0.88
		ESBL	2	2	0.5	0.5	0.12–0.88
	UVRR	CRE	4	0	1	0	0.56–1.00
		ESBL	4	0	0	1	0.00–0.44
PLS-DA	785 nm	CRE	3	1	0.75	0.5	0.28–0.97
		ESBL	2	2	0.5	0.75	0.12–0.88
	UVRR	CRE	2	2	0.5	0	0.12–0.88
		ESBL	4	0	0	0.5	0.00–0.44
PCA-RF	785 nm	CRE	2	2	0.5	0.75	0.12–0.88
		ESBL	1	3	0.75	0.5	0.28–0.97
	UVRR	CRE	0	4	0	0	0.00–0.44
		ESBL	4	0	0	0	0.00–0.44

Table S5C.1. Confusion matrix and 95% confidence intervals (CI) of sensitivity of training data for all machine-learning models for the classification of *K. pneumoniae* susceptible vs resistant strains

		TRUE	PREDICTED				
			Resistant	Susceptible	Sensitivity	Specificity	Sensitivity (95% CI)
PCA-LDA	785 nm	Resistant	9	1	0.9	0.8	0.62–0.99
		Susceptible	1	4	0.8	0.9	0.37–0.98
	UVRR	Resistant	5	5	0.5	0.6	0.22–0.78
		Susceptible	2	3	0.6	0.5	0.21–0.91
PCA-SVM	785 nm	Resistant	10	0	1	0.8	0.78–1.00
		Susceptible	1	4	0.8	1	0.37–0.98
	UVRR	Resistant	1	9	0.1	0.4	0.01–0.38
		Susceptible	3	2	0.4	0.1	0.09–0.79
PLS-DA	785 nm	Resistant	9	1	0.9	1	0.62–0.99
		Susceptible	0	5	1	0.9	0.62–1.00
	UVRR	Resistant	4	6	0.4	0.4	0.15–0.70
		Susceptible	3	2	0.4	0.4	0.09–0.79
PCA-RF	785 nm	Resistant	8	2	0.8	0.2	0.50–0.96
		Susceptible	4	1	0.2	0.8	0.02–0.63
	UVRR	Resistant	10	0	1	0	0.78–1.00
		Susceptible	5	0	0	1	0.00–0.38

Table S5C.2. Confusion matrix and 95% confidence intervals (CI) of sensitivity of training data for all machine-learning models for the classification of *K. pneumoniae* ESBL vs CRE

		TRUE	PREDICTED				
			CRE	ESBL	Sensitivity	Specificity	Sensitivity (95% CI)
PCA-LDA	785 nm	CRE	4	1	0.8	0.8	0.37–0.98
		ESBL	1	4	0.8	0.8	0.37–0.98
	UVRR	CRE	2	3	0.4	0.2	0.09–0.79
		ESBL	4	1	0.2	0.4	0.02–0.63
PCA-SVM	785 nm	CRE	4	1	0.8	0.6	0.37–0.98
		ESBL	2	3	0.6	0.8	0.21–0.91
	UVRR	CRE	3	2	0.6	0	0.21–0.91
		ESBL	5	0	0	0.6	0.00–0.38
PLS-DA	785 nm	CRE	4	1	0.8	0.6	0.37–0.98
		ESBL	2	3	0.6	0.8	0.21–0.91
	UVRR	CRE	0	5	0	0.2	0.00–0.38
		ESBL	4	1	0.2	0	0.02–0.63
PCA-RF	785 nm	CRE	4	1	0.8	0.8	0.67–1.00
		ESBL	1	4	0.8	0.8	0.04–0.82
	UVRR	CRE	2	3	0.4	0.6	0.09–0.79
		ESBL	2	3	0.6	0.4	0.21–0.91

Table S5D. Confusion matrix and 95% confidence intervals (CI) of sensitivity of training data for all machine-learning models for the classification of *K. oxytoca* susceptible vs resistant strains

		TRUE	PREDICTED				
			Resistant	Susceptible	Sensitivity	Specificity	Sensitivity (95% CI)
PCA-LDA	785 nm	Resistant	2	1	0.667	0.667	0.18–0.96
		Susceptible	1	2	0.667	0.667	0.18–0.96
	UVRR	Resistant	1	2	0.333	1	0.04–0.82
		Susceptible	0	3	1	0.333	0.46–1.00
PCA-SVM	785 nm	Resistant	3	0	1	0.333	0.46–1.00
		Susceptible	2	1	0.333	1	0.04–0.82
	UVRR	Resistant	1	2	0.333	1	0.04–0.82
		Susceptible	0	3	1	0.333	0.46–1.00
PLS-DA	785 nm	Resistant	3	0	1	0.333	0.46–1.00
		Susceptible	2	1	0.333	1	0.04–0.82
	UVRR	Resistant	0	3	0	0.333	0.00–0.54
		Susceptible	2	1	0.333	0	0.04–0.82
PCA-RF	785 nm	Resistant	1	2	0.333	0	0.04–0.82
		Susceptible	3	0	0	0.333	0.00–0.54
	UVRR	Resistant	0	3	0	0	0.00–0.54
		Susceptible	3	0	0	0	0.00–0.54

Table S5E.1. Confusion matrix and 95% confidence intervals (CI) of sensitivity of training data for all machine-learning models for the classification of *A. baumanii* susceptible vs resistant strains

		TRUE	PREDICTED				
			Resistant	Susceptible	Sensitivity	Specificity	Sensitivity (95% CI)
PCA-LDA	785 nm	Resistant	5	1	0.833	0.667	0.44–0.98
		Susceptible	1	2	0.667	0.833	0.18–0.96
	UVRR	Resistant	4	2	0.667	1	0.29–0.92
		Susceptible	0	3	1	0.667	0.46–1.00
PCA-SVM	785 nm	Resistant	4	2	0.667	0.667	0.29–0.92
		Susceptible	1	2	0.667	0.667	0.18–0.96
	UVRR	Resistant	2	4	0.333	1	0.04–0.82
		Susceptible	0	3	1	0.333	0.04–0.82
PLS-DA	785 nm	Resistant	3	3	0.5	0.667	0.17–0.83
		Susceptible	1	2	0.667	0.5	0.18–0.96
	UVRR	Resistant	3	3	0.5	1	0.17–0.83
		Susceptible	0	3	1	0.5	0.46–1.00
PCA-RF	785 nm	Resistant	6	0	1	0.333	0.67–1.00
		Susceptible	2	1	0.333	1	0.04–0.82
	UVRR	Resistant	6	0	1	0.333	0.67–1.00
		Susceptible	2	1	0.333	1	0.04–0.82

Table S5E.2. Confusion matrix and 95% confidence intervals (CI) of sensitivity of training data for all machine-learning models for the classification of *A. baumanii* ESBL vs CRE strains

		TRUE	PREDICTED				
			CRE	ESBL	Sensitivity	Specificity	Sensitivity (95% CI)
PCA-LDA	785 nm	CRE	2	1	0.667	0.667	0.18–0.96
		ESBL	1	2	0.667	0.667	0.18–0.96
	UVRR	CRE	1	2	0.333	0.333	0.04–0.82
		ESBL	2	1	0.333	0.333	0.04–0.82
PCA-SVM	785 nm	CRE	3	0	1	0	0.46–1.00
		ESBL	3	0	0	1	0.00–0.54
	UVRR	CRE	1	2	0.333	0.333	0.04–0.82
		ESBL	2	1	0.333	0.333	0.04–0.82
PLS-DA	785 nm	CRE	2	1	0.667	0	0.18–0.96
		ESBL	3	0	0	0.667	0.00–0.54
	UVRR	CRE	2	1	0.667	0.333	0.18–0.96
		ESBL	2	1	0.333	0.667	0.04–0.82
PCA-RF	785 nm	CRE	2	1	0.667	0.333	0.18–0.96
		ESBL	2	1	0.333	0.667	0.04–0.82
	UVRR	CRE	0	3	0	0.333	0.00–0.54
		ESBL	2	1	0.333	0	0.04–0.82

Table S5F. Confusion matrix and 95% confidence intervals (CI) of sensitivity of training data for all machine-learning models for the classification of *C. freundii* susceptible vs resistant strains

		TRUE	PREDICTED				
			Resistant	Susceptible	Sensitivity	Specificity	Sensitivity (95% CI)
PCA-LDA	785 nm	Resistant	2	1	0.667	0.667	0.18–0.96
		Susceptible	1	2	0.667	0.667	0.18–0.96
	UVRR	Resistant	2	1	0.667	0.667	0.18–0.96
		Susceptible	1	2	0.667	0.667	0.18–0.96
PCA-SVM	785 nm	Resistant	2	1	0.667	1	0.18–0.96
		Susceptible	0	3	1	0.667	0.46–1.00
	UVRR	Resistant	1	2	0.333	0.667	0.04–0.82
		Susceptible	1	2	0.667	0.333	0.18–0.96
PLS-DA	785 nm	Resistant	2	1	0.667	0.667	0.18–0.96
		Susceptible	1	2	0.667	0.667	0.18–0.96
	UVRR	Resistant	2	1	0.667	0.333	0.18–0.96
		Susceptible	2	1	0.333	0.667	0.04–0.82
PCA-RF	785 nm	Resistant	2	1	0.667	0	0.18–0.96
		Susceptible	3	0	0	0.667	0.00–0.54
	UVRR	Resistant	0	3	0	0	0.00–0.54
		Susceptible	3	0	0	0	0.00–0.54

Table S5G. Confusion matrix and 95% confidence intervals (CI) of sensitivity of training data for all machine-learning models for the classification of *E. faecium* VSE vs VRE strains

		TRUE	PREDICTED				
			VRE	VSE	Sensitivity	Specificity	Sensitivity (95% CI)
PCA-LDA	785 nm	VRE	2	2	0.5	1	0.12–0.88
		VSE	0	4	1	0.5	0.56–1.00
	UVRR	VRE	3	1	0.75	0.25	0.28–0.97
		VSE	3	1	0.25	0.75	0.03–0.72
PCA-SVM	785 nm	VRE	1	3	0.25	1	0.03–0.72
		VSE	0	4	1	0.25	0.56–1.00
	UVRR	VRE	4	0	1	0.25	0.56–1.00
		VSE	3	1	0.25	1	0.03–0.72
PLS-DA	785 nm	VRE	3	1	0.75	0.5	0.28–0.97
		VSE	2	2	0.5	0.75	0.12–0.88
	UVRR	VRE	2	2	0.5	0.25	0.12–0.88
		VSE	3	1	0.25	0.5	0.03–0.72
PCA-RF	785 nm	VRE	2	2	0.5	0.5	0.12–0.88
		VSE	2	2	0.5	0.5	0.12–0.88
	UVRR	VRE	1	3	0.25	0	0.03–0.72
		VSE	4	0	0	0.25	0.00–0.44

Table S6. Confusion matrix and 95% confidence intervals (CI) of sensitivity of test data for all machine-learning models for the classification of bacterial species

		Test (Balanced Accuracy / %)						
UVRR	*PCA-LDA*	*Mean Sensitivity (95% CI)*	*PCA-SVM*	*Mean Sensitivity (95% CI)*	*PLS-DA*	*Mean Sensitivity (95% CI)*	*PCA-RF*	*Mean Sensitivity (95% CI)*
	66.7	0.47–0.75	*72.2*	0.51–0.80	*58.3*	0.41–0.71	*58.3*	0.41–0.71
785 nm	*PCA-LDA*		*PCA-SVM*		*PLS-DA*		*PCA-RF*	
	100	0.70–0.96	*94.4*	*0.61–0.90*	*94.4*	*0.65–0.94*	*86.1*	*0.59–0.90*

Table S7. Test results and 95% confidence intervals (CI) of sensitivity of susceptible vs. resistant strains for each bacterial species

		Test (Balanced Accuracy / %)							
		PCA-LDA	Mean Sensitivity (95% CI)	PCA-SVM	Mean Sensitivity (95% CI)	PLS-DA	Mean Sensitivity (95% CI)	PCA-RF	Mean Sensitivity (95% CI)
UVRR	A. baumanii	50	0.23–0.88	50	0.23–0.88	50	0.23–0.88	25	
	C. freundii	100	0.35–0.99	100	0.35–0.99	50	0.14–0.86	100	0.35–0.99
	E. faecium	0	0.01–0.65	50	0.14–0.86	0	0.01–0.65	50	0.14–0.86
	E. coli	75	0.24–0.93	75	0.24–0.93	100	0.44–0.99	50	0.23–0.88
	K. oxytoca	0	0.01–0.65	0	0.01–0.65	50	0.14–0.86	50	0.14–0.86
	K. pneumoniae	50	0.23–0.88	25	0.07–0.76	25	0.07–0.76	50	0.23–0.88
		PCA-LDA		PCA-SVM		PLS-DA		PCA-RF	
785 nm	A. baumanii	50	0.12–0.77	50	0.12–0.77	50	0.12–0.77	50	0.12–0.77
	C. freundii	50	0.14–0.86	50	0.14–0.86	50	0.14–0.86	0	0.01–0.65
	E. faecium	0	0.01–0.65	50	0.14–0.86	50	0.14–0.86	50	0.14–0.86
	E. coli	50	0.12–0.77	75	0.24–0.93	50	0.12–0.77	75	0.24–0.93
	K. oxytoca	0	0.01–0.65	100	0.35–0.99	100	0.35–0.99	100	0.35–0.99
	K. pneumoniae	100	0.44–0.99	100	0.44–0.99	100	0.44–0.99	100	0.44–0.99

Table S8. Test results and 95% confidence intervals (CI) of sensitivity of ESBL vs. CRE strains for each bacterial species

		Test (Balanced Accuracy / %)							
		PCA-LDA	*Mean Sensitivity (95% CI)*	*PCA-SVM*	*Mean Sensitivity (95% CI)*	*PLS-DA*	*Mean Sensitivity (95% CI)*	*PCA-RF*	*Mean Sensitivity (95% CI)*
UVRR	*A. baumanii*	*50*	0.14–0.86	*100*	0.35–0.99	*50*	0.14–0.86	*50*	0.14–0.86
	E. coli	*50*	0.14–0.86	*0*	0.01–0.65	*50*	0.14–0.86	*0*	0.01–0.65
	K. pneumoniae	*50*	0.14–0.86	*50*	0.14–0.86	*50*	0.14–0.86	*50*	0.14–0.86
		PCA-LDA		*PCA-SVM*		*PLS-DA*		*PCA-RF*	
785 nm	*A. baumanii*	*50*	*0.31–0.89*	*100*	*0.35–0.99*	*100*	*0.35–0.99*	*100*	*0.35–0.99*
	E. coli	*100*	*0.35–0.99*	*100*	*0.35–0.99*	*100*	*0.35–0.99*	*50*	*0.14–0.86*
	K. pneumoniae	*100*	*0.35–0.99*	*100*	*0.35–0.99*	*100*	*0.35–0.99*	*50*	*0.14–0.86*

References

(1) Neugebauer, U.; Clement, J. H.; Bocklitz, T.; Krafft, C.; Popp, J. Identification and differentiation of single cells from peripheral blood by Raman spectroscopic imaging. *Journal of Biophotonics* **2010**, *3* (8-9), 579-587. DOI: 10.1002/jbio.201000020.

(2) Neugebauer, U.; Schmid, U.; Baumann, K.; Simon, H.; Schmitt, M.; Popp, J. DNA tertiary structure and changes in DNA supercoiling upon interaction with ethidium bromide and gyrase monitored by UV resonance Raman spectroscopy. *Journal of Raman Spectroscopy* **2007**, *38* (10), 1246-1258. DOI: 10.1002/jrs.1760.

(3) Walter, A.; Reinicke, M.; Bocklitz, T.; Schumacher, W.; Rösch, P.; Kothe, E.; Popp, J. Raman spectroscopic detection of physiology changes in plasmid-bearing Escherichia coli with and without antibiotic treatment. *Anal Bioanal Chem* **2011**, *400* (9), 2763-2773. DOI: 10.1007/s00216-011-4819-4 From NLM.

(4) Harz, M.; Claus, R. A.; Bockmeyer, C. L.; Baum, M.; Rösch, P.; Kentouche, K.; Deigner, H. P.; Popp, J. UV-resonance Raman spectroscopic study of human plasma of healthy donors and patients with thrombotic microangiopathy. *Biopolymers* **2006**, *82* (4), 317-324. DOI: 10.1002/bip.20489 From NLM.

(5) Shanmugasundaram, M.; Puranik, M. Computational prediction of vibrational spectra of normal and modified DNA nucleobases. *Journal of Raman Spectroscopy* **2009**, *40* (12), 1726-1748. DOI: 10.1002/jrs.2533.

(6) Czamara, K.; Majzner, K.; Pacia, M. Z.; Kochan, K.; Kaczor, A.; Baranska, M. Raman spectroscopy of lipids: a review. *Journal of Raman Spectroscopy* **2015**, *46* (1), 4-20. DOI: 10.1002/jrs.4607.

(7) Maquelin, K.; Kirschner, C.; Choo-Smith, L. P.; van den Braak, N.; Endtz, H. P.; Naumann, D.; Puppels, G. J. Identification of medically relevant microorganisms by vibrational spectroscopy. *J Microbiol Methods* **2002**, *51* (3), 255-271. DOI: 10.1016/s0167-7012(02)00127-6.

(8) Germond, A.; Ichimura, T.; Horinouchi, T.; Fujita, H.; Furusawa, C.; Watanabe, T. M. Raman spectral signature reflects transcriptomic features of antibiotic resistance in Escherichia coli. *Communications Biology* **2018**, *1* (1), 85. DOI: 10.1038/s42003-018-0093-8.

(9) Notingher, I.; Hench, L. L. Raman microspectroscopy: a noninvasive tool for studies of individual living cells in vitro. *Expert Review of Medical Devices* **2006**, *3* (2), 215-234. DOI: 10.1586/17434440.3.2.215.

(10) Huang, W. E.; Li, M.; Jarvis, R. M.; Goodacre, R.; Banwart, S. A. Chapter 5 - Shining Light on the Microbial World: The Application of Raman Microspectroscopy. In *Advances in Applied Microbiology*, Vol. 70; Academic Press, 2010; pp 153-186.

(11) Benevides, J. M.; Overman, S. A.; Thomas Jr, G. J. Raman, polarized Raman and ultraviolet resonance Raman spectroscopy of nucleic acids and their complexes. *Journal of Raman Spectroscopy* **2005**, *36* (4), 279-299. DOI: 10.1002/jrs.1324.

(12) Harz, M.; Krause, M.; Bartels, T.; Cramer, K.; Rösch, P.; Popp, J. Minimal Invasive Gender Determination of Birds by Means of UV-Resonance Raman Spectroscopy. *Analytical Chemistry* **2008**, *80* (4), 1080-1086. DOI: 10.1021/ac702043q.

(13) Pistiki, A.; Ramoji, A.; Ryabchykov, O.; Thomas-Rüddel, D.; Press, A. T.; Makarewicz, O.; Giamarellos-Bourboulis, E. J.; Bauer, M.; Bocklitz, T.; Popp, J.; et al. Biochemical Analysis of Leukocytes after In Vitro and In Vivo Activation with Bacterial and Fungal Pathogens Using Raman Spectroscopy. *International Journal of Molecular Sciences* **2021**, *22* (19). DOI: 10.3390/ijms221910481.

(14) Rygula, A.; Majzner, K.; Marzec, K. M.; Kaczor, A.; Pilarczyk, M.; Baranska, M. Raman spectroscopy of proteins: a review. *Journal of Raman Spectroscopy* **2013**, *44* (8), 1061-1076. DOI: 10.1002/jrs.4335.

(15) Teng, L.; Wang, X.; Wang, X.; Gou, H.; Ren, L.; Wang, T.; Wang, Y.; Ji, Y.; Huang, W. E.; Xu, J. Label-free, rapid and quantitative phenotyping of stress response in E. coli via ramanome. *Scientific Reports* **2016**, *6* (1), 34359. DOI: 10.1038/srep34359.
